# An interpretable machine learning model for predicting 28-day mortality in patients with sepsis-associated liver injury

**DOI:** 10.1371/journal.pone.0303469

**Published:** 2024-05-20

**Authors:** Chengli Wen, Xu Zhang, Yong Li, Wanmeng Xiao, Qinxue Hu, Xianying Lei, Tao Xu, Sicheng Liang, Xiaolan Gao, Chao Zhang, Zehui Yu, Muhan Lü

**Affiliations:** 1 Department of Intensive Care Medicine, Department of Critical Care Medicine, The Affiliated Hospital, Southwest Medical University, Luzhou, China; 2 Luzhou Key Laboratory of Human Microecology and Precision Diagnosis and Treatment, Luzhou, China; 3 Southwest Medical University, Luzhou, China; 4 Department of Gastroenterology, The Affiliated Hospital, Southwest Medical University, Luzhou, China; 5 Laboratory Animal Center, Southwest Medical University, Luzhou, China; Mae Fah Luang University, THAILAND

## Abstract

Sepsis-Associated Liver Injury (SALI) is an independent risk factor for death from sepsis. The aim of this study was to develop an interpretable machine learning model for early prediction of 28-day mortality in patients with SALI. Data from the Medical Information Mart for Intensive Care (MIMIC-IV, v2.2, MIMIC-III, v1.4) were used in this study. The study cohort from MIMIC-IV was randomized to the training set (0.7) and the internal validation set (0.3), with MIMIC-III (2001 to 2008) as external validation. The features with more than 20% missing values were deleted and the remaining features were multiple interpolated. Lasso-CV that lasso linear model with iterative fitting along a regularization path in which the best model is selected by cross-validation was used to select important features for model development. Eight machine learning models including Random Forest (RF), Logistic Regression, Decision Tree, Extreme Gradient Boost (XGBoost), K Nearest Neighbor, Support Vector Machine, Generalized Linear Models in which the best model is selected by cross-validation (CV_glmnet), and Linear Discriminant Analysis (LDA) were developed. Shapley additive interpretation (SHAP) was used to improve the interpretability of the optimal model. At last, a total of 1043 patients were included, of whom 710 were from MIMIC-IV and 333 from MIMIC-III. Twenty-four clinically relevant parameters were selected for model construction. For the prediction of 28-day mortality of SALI in the internal validation set, the area under the curve (AUC (95% CI)) of RF was 0.79 (95% CI: 0.73–0.86), and which performed the best. Compared with the traditional disease severity scores including Oxford Acute Severity of Illness Score (OASIS), Sequential Organ Failure Assessment (SOFA), Simplified Acute Physiology Score II (SAPS II), Logistic Organ Dysfunction Score (LODS), Systemic Inflammatory Response Syndrome (SIRS), and Acute Physiology Score III (APS III), RF also had the best performance. SHAP analysis found that Urine output, Charlson Comorbidity Index (CCI), minimal Glasgow Coma Scale (GCS_min), blood urea nitrogen (BUN) and admission_age were the five most important features affecting RF model. Therefore, RF has good predictive ability for 28-day mortality prediction in SALI. Urine output, CCI, GCS_min, BUN and age at admission(admission_age) within 24 h after intensive care unit(ICU) admission contribute significantly to model prediction.

## Introduction

Sepsis is a syndrome of multiple organ dysfunction caused by an abnormal immune response to infection [1], and being one of the common diseases in intensive care units (ICU), it has been an important global health problem. The Global Burden of Disease Study, published in 2020, analyzed global, regional and national sepsis incidence and mortality rates from 1990 to 2017 and reported that there were approximately 48.9 million cases of sepsis in 2017, with about 11 million sepsis-related deaths, accounting for 19.7% of all deaths worldwide [2]. High risk of rehospitalization and high cost of treatment for sepsis [3,4]. In the United States, sepsis was the most expensive condition treated, amounting to $38.2 billion or 8.8% of aggregate costs for all hospital stays in 2017 [5].

The liver is a vital organ for the human body which regulates the balance of metabolism and immunity [6,7]. The liver is essential for regulating immune defense during sepsis and the mechanisms it is involved with are lipopolysaccharide detoxification, bacterial clearance, acute-phase protein and cytokine release, inflammation metabolic regulation, etc. [8] The production of large amounts of endotoxins and the release of inflammatory factors in sepsis lead to abnormal immune responses that impair the function of multiple organs, including the liver [9]. When there is an inappropriate immune response or excessive inflammation in the liver, the ability to clear pathogens is impaired and liver metabolism is disrupted. Sepsis associated liver injury (SALI) can be caused by a variety of factors, including pathogens or shock, an exaggerated inflammatory response, persistent microcirculation failure, or even oxidative stress [10]. There are two main manifestations of SALI: ischemic hypoxic liver injury and sepsis-related cholestasis. There are no unified diagnostic criteria for SALI, and the Surviving Sepsis Campaign (SSC) Guidelines recommended to use total bilirubin(TBIL) >2 mg/dL and international standardized ratio (INR) >1.5 as the diagnostic criteria [11]. In the assessment of the severity of disease, Sequential Organ Failure Assessment (SOFA) [12], Oxford Acute Severity of Illness Score (OASIS) [13], Acute Physiology Score III (APS III) [14], Logistic Organ Dysfunction Score (LODS) [15], Simplified Acute Physiology Score II (SAPS II) [16], Systemic Inflammatory Response Syndrome (SIRS), and Glasgow Coma Scale (GCS) were some traditional scorings of disease severity.

Studies have shown that the incidence of SALI in the U.S. adult sepsis population is 34% and 46%, which is considered as an independent risk factor for death from sepsis, and that patients who develop SALI have an increased risk of death of nearly 54% [9,17]. The high mortality rate of SALI may be related to the lack of effective diagnostic tools and early warning systems. The aim of this study is to develop an explicable machine learning model that can predict the 28-day mortality of SALI early, provide early warning for SALI, and remind clinicians to conduct effective clinical interventions in patients to reduce their 28-day mortality.

## Methods

### Data source

This is a retrospective cohort study based on data the extracted from two open databases at the same center, including critical care databases v2.2 (Medical Information Mart for Intensive Care (MIMIC-IV)) (2008 to 2019) and v1.4 (MIMIC-III) (2001 to 2008) collected from Beth Israel Deaconess Medical Center in Boston. We were granted access to the database (Chengli Wen ID 11718300).

### Research population

We included patients ≥18 years old with sepsis which was defined as infection with a SOFA score ≥2 according to the sepsis 3.0 diagnostic criteria [1], with an ICU stay ≥24 h and at least one occurrence of SALI (SALI is defined as TBIL>2 mg/dL and INR >1.5 in sepsis [11]). We excluded patients aged <18 years, without liver injury, with ICU stay <24h, and all patients with other types of liver disease. Patients with human immunodeficiency virus (HIV) infection, pregnant women, and patients without biochemical and coagulation tests within 24h of admission to the ICU were also excluded.

### Data collection

We used Structured Query Language (SQL, version 15.1) to extract data from the two databases. To develop optimal early predictive interpretable machine learning model for 28-day mortality in patients with SALI, we extracted seven types of data and 79 candidate clinical features. We retrospectively collected the following data: (1) demographic characteristics, including age, sex, body weight, body height, and body mass index (BMI); (2) medical history which was obtained according to the International Classification of Diseases (ICD)-9 and ICD-10, including hypertension, diabetes, congestive heart failure, myocardial infarction, peptic ulcer, cerebrovascular disease, chronic obstructive pulmonary disease, kidney disease, and Charlson Comorbidity Index (CCI) [18]; (3) vital signs, including heart rate, systolic blood pressure (SBP), diastolic blood pressure (DBP), mean arterial pressure (MAP), respiratory rate(RR), body temperature, and oxygen saturation (SPO_2_); (4) laboratory parameters, including white blood cell count, neutrophils, lymphocytes, platelets, hematocrit, red blood cell distribution width (RDW), hemoglobin, Hypersensitive c-reactive protein (hs-CRP), activated partial thromboplastin time (APTT), prothrombin time (PT), partial thromboplastin time (PTT), INR, fibrinogen, alanine aminotransferase (ALT), aspartate aminotransferase (AST), alkaline phosphatase (ALP), amylase, TBIL, lactate dehydrogenase (LDH), albumin, triglyceride, high-density lipoprotein, low-density lipoprotein, blood urea nitrogen (BUN), serum creatinine(Cr), creatine phosphokinase, creatine kinase MB, high sensitivity troponin T, N-terminal pro brain natriuretic peptide (NT-pro-BNP), lactate, pH, pO_2_, pCO_2_, PaO_2_/FiO_2_ ratio, base excess, anion gap, bicarbonate, serum calcium, serum chloride, serum sodium, serum potassium, and blood glucose; (5) traditional scores for assessing disease severity, including OASIS, SOFA, SIRS, SAPS II, LODS, GCS, and APSIII; and (6) urine volume on the first day of ICU admission; and (7) others, including duration of ICU stay this time, infection site, dopamine (ug/kg. min), adrenalin (ug/kg. min), and noradrenaline (ug/kg. min), dobutamine (ug/kg. min). The 28-day mortality rate was an outcome indicator. A detailed list of the included variables is shown in S1 Table.

### Ethics statements

The databases were approved by the Massachusetts Institute of Technology and Beth Israel Deaconess Medical Center. This study is a retrospective study and does not affect clinical treatment and care; Therefore, the ethical approval statement and informed consent of each patient included in the study were waived [19]. This study is consistent with the Transparent Reporting of Multivariate Predictive Models for Individual Prognosis or Diagnosis (TRIPOD): TRIPOD statement [20], and the TRIPOD checklist showed in S2 Table.

### Data preprocessing

All data processing was done in the R or python environment. First, the cohorts from the two databases were divided into either the death group or the survival group (Patient outcome defined as 1 for death and 0 for survival), and the differences in each of the clinical features between the two group were compared. Second, we conducted a missing value analysis (S3 Table) and removed features with missing values exceeding 20%. Third, we used multiple interpolation to interpolate features with less than 20% missing values. The data overlap before and after interpolation were good, and the distribution of the original and interpolated data is shown in S1 Fig. Then, based on the Lasso-CV method with an optimal regularization parameter of 0.113 for feature screening of the interpolated data after excluding SIRS, SOFA, OASIS, SAPSII, LODS, and APSIII, which are comprehensive scores that can comprehensive assessment the severity of the disease, 24 features were ultimately selected to develop the model (S4 Table).

### Model development and validation

The data extracted from MIMIC-IV were randomly divided into training and internal validation sets according to 7:3, and the data from MIMIC-III that did not overlap with MIMIC-IV were used as the external validation set. We chose the following eight models in the training set for model training: Random Forest (RF), Logistic Regression, Decision Tree, Extreme Gradient Boost (XGBoost), K Nearest Neighbor Model (KNN), Support Vector Machine (SVM), Network for Generalized Linear Models in which the best model is selected by cross-validation (CV_glmnet), and Linear Discriminant Analysis (LDA). Internal and external validation sets were used to test the performance of the model. We used area under the curve (AUC), accuracy, precision, recall, and specificity to evaluate the performance of the models, and the most important of these indicators was AUC. The optimal model was compared with the traditional clinical disease severity scores (SIRS, SOFA, OASIS, SAPSII, LODS, and APSIII) to better predict the 28-day mortality risk of patients with SALI, in order to alert the clinicians to make early interventions. We hyper-parameterized the optimal model to obtain the optimal performance of the model.

### Model explainability

The Shapley additive explanations (SHAP) method was used to improve the interpretability of the final model. SHAP is a machine learning interpretation method that can be used to interpret the importance of features in model prediction results [21]. It is based on the concept of Shapley value in cooperative game theory and uses an additive method to calculate the contribution of each feature to the model prediction results. The SHAP algorithm can provide an explanatory value for each feature, indicating the degree of influence of the feature on the model’s prediction results, and the results of the calculation can be used to explain not only the feature importance of individual predictions, but also the feature importance distribution of the entire dataset.

### Statistical analysis

Values are expressed as medians (interquartile range) for continuous variables and totals (percentages) for categorical variables. The rank sum test was used for continuous variables and the Chi-square test for categorical variables. After data preprocessing and feature selection, we developed eight popular machine learning models to predict 28-day mortality in patients with sepsis-related liver injury. The overall performance of each model was evaluated on their AUC, accuracy, precision, recall, and specificity. The best performing model was interpreted using Shapley values.

All calculations and analyses were performed using R 4.2.1 and Python 3.7 software. All statistical tests were 2-sided, and P values<0.05 were considered to be statistically significant.

## Results

### Baseline characteristics

**Fig 1:**
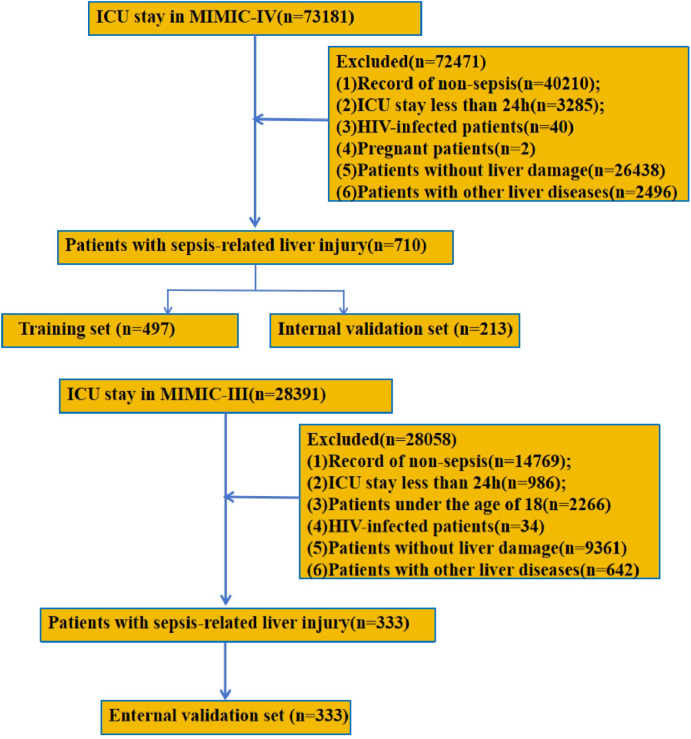
The flow chart of this study. **A.** Screening Process for MIMIC-IV. **B.** Screening Process for MIMIC-III.

There was a total of 73,181 records in MIMIC-IV, and after screening the records based on the inclusion and exclusion criteria, 710 records were finally obtained. Of these, 497 cases were used as the training set and 213 cases were used as the internal validation set. MIMIC-III (2001–2008) included 28,391 records, with 333 patients ultimately included as an external validation set. The flow chart of this study is shown in Fig 1. Table 1 shows the baseline characteristics of the entire cohort from MIMIC-IV, as well as the death and survival groups. Baseline characteristics of the cohort from MIMIC-III are shown in S5 Table. The cohort from MIMIC-IV included 404 male (56.9%) and 306 female (43.1%), with 424 survivors (59.7%) and 286 deaths (40.3%), and the median (interquartile range [IQR]) age was 68.4 (57.5,78.8) years. The age of patients in the death group (70.6[62,80.9]) was significantly higher than that in the survival group (66.1[54.2,77.2]). Compared to the survival group, the death group had a higher CCI (6.0[4.0,7.0] vs 7.0[5.0,9.0], P<0.001) and more patients with diabetes (97(33.9%) vs 128(30.2%), P<0.001) and Myocardial infarction (67(15.8%) vs 70(24.5%), P = 0.022). In addition, among the laboratory test indices, RDW (15.7[14.2,17.7] vs 16.6[14.7,18.6], P<0.001), ALP (15.7[14.2,17.7] vs 99[62,178.8], P<0.001), LDH (338.0[236.0,521.0] vs 440.5[283.3,792.2], P<0.001), BUN (25.0[16.0,40.0] vs 36.0[22.0,57.0], P<0.001), Cr (1.3[0.9,1.9] vs 25.0[16.0,40.0], P = 0.001), and Lactate (2.1[1.4,3.4] vs 2.3[1.5,4.0], P<0.001) were higher in the death group, while pO_2_ (134[86,254] vs 112.5[74.3,206.5], P = 0.006), Albumin (3.0[2.5,3.4] vs 2.7[2.2,3.2], P<0.001), Platelets (163.0[101.5,228.0] vs 139.0[74.0,211.0], P = 0.002), and Hemoglobin (139.0[74.0,211.0] vs 10.0[8.5,12.2], P = 0.032) were lower. We also found that the survival group had a longer ICU stay (6.2[3.0,13.0] vs 4.5[2.4,7.7], P<0.001) and 24h urine output (1500[878,2270] vs 382[780,1650], P<0.001) than the death group. All of the scores for disease severity, except for the SIRS score, were significantly higher for the death group than the survival group.

**Table 1:** Baseline characteristics of the cohort from MIMIC-IV.

Characteristics	All(N = 710)	Survival(N = 424)	Non-survival(N = 286)	P value
**Demographic**				
Age, year	68.4(57.5,78.8)	66.1(54.2,77.2)	70.6(62,80.9)	<0.001
Sex				0.034
Male, n (%)	404(56.9)	255(60.1)	149(52.1)	
Female, n (%)	306(43.1)	169(39.9)	137(47.9)	
Body weight, kg	80.6(66.0,96.0)	82(67.2,98.6)	78.3(64.5,93)	0.018
Height, cm BMI, kg/㎡	168.9(160,178) 27.4(24.0,31.3)	170(160,178) 27.3(24.2,31.3)	168.0(161.0,175.0) 27.7(23.4,31.7)	0.030 0.403
**Comorbidities**				
Charlson Comorbidity Index	6.0(4.0,8.0)	6.0(4.0,7.0)	7.0(5.0,9.0)	<0.001
Hypertension,n(%)	462(65.1)	244(57.5)	218(76.2)	0.432
Diabetes,n(%)	225(31.7)	128(30.2)	97(33.9)	<0.001
Congestive heart failure,n(%)	312(43.9)	181(42.7)	131(45.8)	0.412
Myocardial infarction,n(%)	137(19.3)	67(15.8)	70(24.5)	0.022
Peptic ulcer,n(%)	34(4.8)	20(4.7)	14(4.9)	0.913
Cerebrovascular disease,n(%)	80(11.3)	50(11.8)	30(10.5)	0.590
Chronic pulmonary disease,n(%)	169(23.8)	98(23.1)	71(24.8)	0.599
Renal disease,n(%)	214(30.1)	120(28.3)	94(32.9)	0.194
**Vital signs on day 1**				
Heart rate, bpm	96(82,111)	94(81,109)	98(85,112)	0.082
Systolic blood pressure, mmHg	109(96,128)	110(96,129)	108(95,126)	0.401
Diastolic blood pressure, mmHg	64(53,76)	64(53,75)	63(53,78)	0.624
Mean arterial pressure, mmHg	75(64,89)	76(64,87)	74(64,91)	0.758
Respiratory rate,	20(16,25)	20(16,25)	21(18,26)	0.023
Body temperature, °C	36.7(36.3,37.2)	36.8(36.4,37.2)	36.7(36.2,37.1)	0.010
SpO_2_, %	98(95,100)	98(96,100)	98(95,100)	0.094
**Laboratory findings on day 1**				
White blood cell, ×10^3^/uL	11.3(7.1,16.9)	11.4(7.4,17.4)	11.1(6.7,15.9)	0.247
Neutrophil, %	82.0(71.2,88.5)	82.0(72.0,88.1)	82.0(69.6,89.0)	0.764
Lymphocyte, %	8.0(4.0,15.6)	8.0(4.0,15.2)	7.7(4.0,15.9)	0.942
Red blood cell distribution width	16.1(14.5,18.2)	15.7(14.2,17.7)	16.6(14.7,18.6)	<0.001
Platelets, ×10^3^/uL	154.5(92.0,224.2)	163.0(101.5,228.0)	139.0(74.0,211.0)	0.002
Hematocrit, %	30.5(26.3,36.2)	30.8(26.5,37)	30.2(26.0,35.3)	0.202
Hemoglobin,g/dL	9.9(8.4,11.9)	10.0(8.5,12.2)	9.6(8.3,11.5)	0.032
Hypersensitivec-reactive protein, mg/L	203.4(113.9,246.4)	231.0(203.4,260.0)	123.7(103.0,220.6)	
D_dimer, ng/mL	5774(3198.0,7301.5)	4626.5(2798.3,5850.5)	8433(5660,8614)	0.400
Prothrombin Time, s	18.2(15.3,22.8)	18.0(15.4,21.9)	28.5(15.2,25.8)	0.294
International normalized ratio Partial thromboplastin time, s	1.7(1.4,2.1) 36.6(30.7,47.3)	1.7(1.4,2.0) 35.7(30.5,46.2)	1.7(1.4,2.4) 37.2(31.5,49.8)	0.276 0.101
Fibrinogen, mg/dL	257(165,407)	251(166.5,401)	288(163,433)	0.647
Alanine aminotransferase, U/L	40.0(21.0,104.0)	43.0(21.0,114.0)	38.0(21.5,84.0)	0.423
Alkaline phosphatase, U/L	110.5(67.8,194.0)	99(62,178.8)	126.0(79.5,212.0)	<0.001
Aspartate aminotransferase, U/L	75(37.8,168.5)	75(36,174)	76(41,163)	0.924
Amylase, U/L	46(33,125)	46(34,107)	49.5(28.8,171.8)	0.953
Total bilirubin, mg/dL	2.9(2.1,4.4)	3.0(2.2,4.3)	2.8(1.7,4.6)	0.144
Lactate dehydrogenase, U/L	362.0(252.0,603.0)	338.0(236.0,521.0)	440.5(283.3,792.2)	<0.001
Albumin, g/L	2.8(2.4,3.4)	3.0(2.5,3.4)	2.7(2.2,3.2)	<0.001
ABLI	0.07(-0.06,0.19)	0.09(-0.02,0.20)	0.07(-0.06,0.20)	0.469
Triglyceride, mg/dL	119.0(83.0,198.5)	151.5(103.5,244.3)	92.5(78.5,112.3)	0.034
High-density lipoprotein, mg/dL	31.0(21.0,38.0)	34.0(22.8,37.8)	31.0(23.0,42.0)	0.830
Low-density lipoprotein, mg/dL	57.0(41.5,86.5)	59.0(42.0,83.0)	55.0(42.5,78.5)	0.755
Blood urea nitrogen, mg/dL	28.0(18.0,48.0)	25.0(16.0,40.0)	36.0(22.0,57.0)	<0.001
Serum creatinine, mg/dL	1.4(0.9,2.1)	1.3(0.9,1.9)	1.5(1.0,2.4)	0.001
Creatine phosphokinase, U/L	137.0(60.0,494.0)	137.5(65.0,390.8)	128.0(59.0,521.0)	0.927
Creatine kinase MB, U/L	4.0(3.0,10.0)	4.0(2.0,8.0)	5.0(3.0,11.5)	0.302
High sensitivity troponin T, ug/L	0.07(0.03,0.27)	0.07(0.03,0.23)	0.08(0.03,0.29)	0.849
N-terminal pro brain natriuretic peptide, pg/mL	6898(2386,17547)	6870.5(1944.3,21520.3)	7934(2586,14921)	0.974
Lactate, mmol/L	2.3(1.5,4.0)	2.1(1.4,3.4)	2.3(1.5,4.0)	<0.001
pH	7.36(7.28,7.44)	7.36(7.29,7.43)	7.36(7.26,7.44)	0.589
pO_2,_ mmHg	126(81,247)	134(86,254)	112.5(74.3,206.5)	0.006
pCO_2_, mmHg	38(32.45)	39(33,45)	37(32,45)	0.187
PaO_2_/FiO_2_ ratio	225.5(135.2,323.)	240(146.3,345.9)	193.8(123.5,308.6)	0.061
Base excess, mmol/L	-2(-6,0)	-2(-6,1)	-2(-8,0)	0.106
Anion gap, mmol/L	17(14,20)	16(13,19)	17(15,20)	<0.001
Bicarbonate, mmol/L	21(18,24)	21(18,25)	21(17,24)	0.079
Serum calcium, mmol/L	8.1(7.6,8.7)	8.2(7.7,8.7)	8.1(7.6,8.7)	0.205
Serum chloride, mmol/L	102(97,107)	103(98,108)	101(96,106)	0.004
Serum sodium, mmol/L	138(134,141)	138.0(134.0,140.5)	137(133,141)	0.170
Serum potassium, mmol/L	4.3(3.7,4.8)	4.2(3.7,4.7)	4.3(3.7,4.9)	0.288
Blood glucose, mg/dL	130(103,167)	128(105,161)	133(100.0,176.0)	0.599
**Others**				
Duration of ICU stay this time, day Site of infection	5.2(2.7,10.9)	6.2(3.0,13.0)	4.5(2.4,7.7)	<0.001
Intestinal, n(%)	50(7.0)	33(7.8)	17(5.9)	0.348
Urinary, n(%)	117(16.5)	72(17.0)	45(15.7)	0.661
Lung, n(%)	162(22.8)	90(21.2)	72(25.2)	0.219
catheter_related, n(%)	40(5.6)	25(5.9)	15(5.2)	0.712
skin_and_soft_tissue, n (%)	57(8.0)	33(7.8)	24(8.4)	0.770
abdominal_cavity, n (%)	94(13.2)	72(17.0)	22(7.7)	<0.001
Dopamine (ug/kg. min)	10.01(5.00,15.03)	10.02(5.00,15.00)	10.01(5.00,15.04)	0.950
Adrenalin (ug/kg. min)	0.07(0.04,0.13)	0.06(0.04,0.10)	0.12(0.07,0.27)	<0.001
Noradrenaline (ug/kg. min)	0.25(0.12,0.45)	0.20(0.10,0.40)	0.30(0.16,0.50)	<0.001
Dobutamine (ug/kg. min)	5.00(3.00,7,51)	5.00(3.75,5.54)	5.01(2.50,7.52)	0.753
**Urine output on day 1, mL**	1221(613,2026)	1500(878,2270)	382(780,1650)	<0.001
**Severity of illness scores**				
GCS SIRS SOFA OASIS	13(8,14) 3(3,4) 10(7,13) 39(32,46))	13(9,14) 3(3,4) 9(7,12) 37(31,44)	11(6,14) 3(3,4) 11(8,14) 42(36,50)	0.021 0.195 <0.001 <0.001
SAPSII	47(38,58)	43(35,52)	54(44,63)	<0.001
LODS	8(6,11)	7(5,10)	10(7,12)	<0.001
APSIII	72(56,94)	64(50,85)	82(67,105)	<0.001

ABLI: Albumin-bilirubin.

### Model development and validation

**Fig 2:**
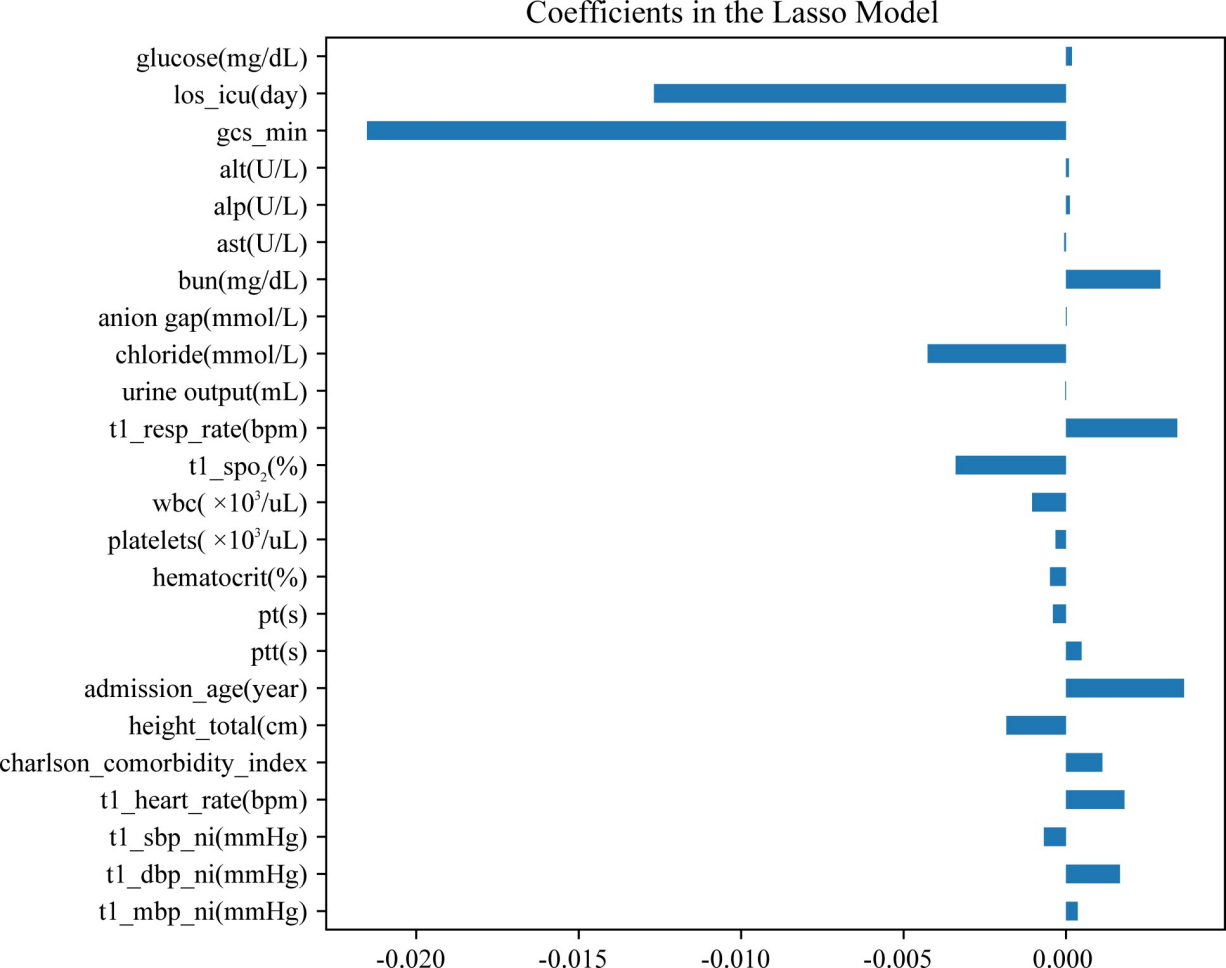
Feature coefficients.

Twenty-four features were screened for model construction (S4 Table). The features coefficients were plotted in Fig 2. A positive value of the coefficient of identity indicates a positive effect on 28-day mortality, while a negative value of the coefficient indicates a negative effect.

**Fig 3:**
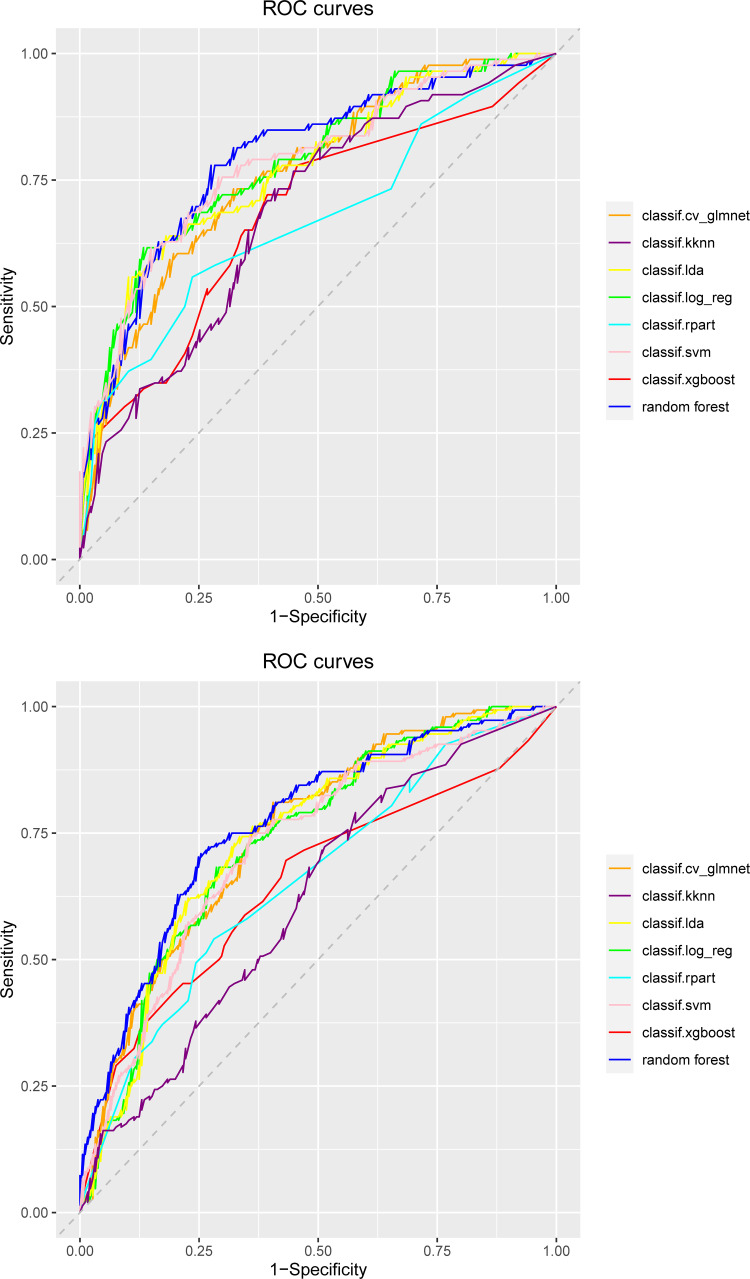
ROC curves of the predictive model. **A.** Internal validation set. **B.** External validation set.

Both internal and external validation sets were used to evaluate the model. In the internal validation cohort, the RF model had good predictive power in predicting sepsis-related liver injury 28-day mortality, with a maximum AUC. 0.79 (95% CI: 0.73–0.86), as compared to CV_glmnet (AUC. 0.76 (95% CI: 0.70–0.83)), Support Vector Machine (AUC. 0.78 (95% CI: 0.72–0.85)), Logistic Regression (AUC. 0.78 (95% CI: 0.70–0.83%)), LDA (AUC. 0.77 (95% CI: 0.70–0.84)), K Nearest Neighbor Model (AUC. 0.69 (95% CI: 0.61–0.76)), XGBoost (AUC. 0.68 (95% CI: 0.61–0.76)), and Decision Tree (AUC. 0.67 (95% CI: 0.59–0.75)). Receiver Operating Characteristic (ROC) were plotted to evaluate the performance of the models, and the ROC curves for the internal validation set and the external validation set are shown in Fig 3. The AUC, accuracy, Precision, Recall, Specificity of the eight models constructed were compared in Table 2.

**Table 2:** Compared performance evaluation of 8 machine learning classification models in predicting 28-day mortality rate in the internal validation set.

Classifiers	AUC	Accuracy (%)	Precision (%)		Recall	Specificity (%)
**random forest**	0.79	74.65	75.52	0.85		59.30
**classif.cv_glmnet** **classif.svm**	0.76 0.78	69.95 72.30	69.57 75.76	0.88 0.79		43.02 62.79
**classif.log_reg**	0.78	74.65	76.26	0.83		61.63
**classif.lda**	0.77	73.24	75.00	0.83		59.30
**classif.kknn**	0.69	61.03	66.92	0.69		50.00
**Classif.xgboost**	0.68	65.26	69.92	0.73		53.48
**classif.rpart**	0.67	66.67	69.72	0.78		50.00

**Fig 4:**
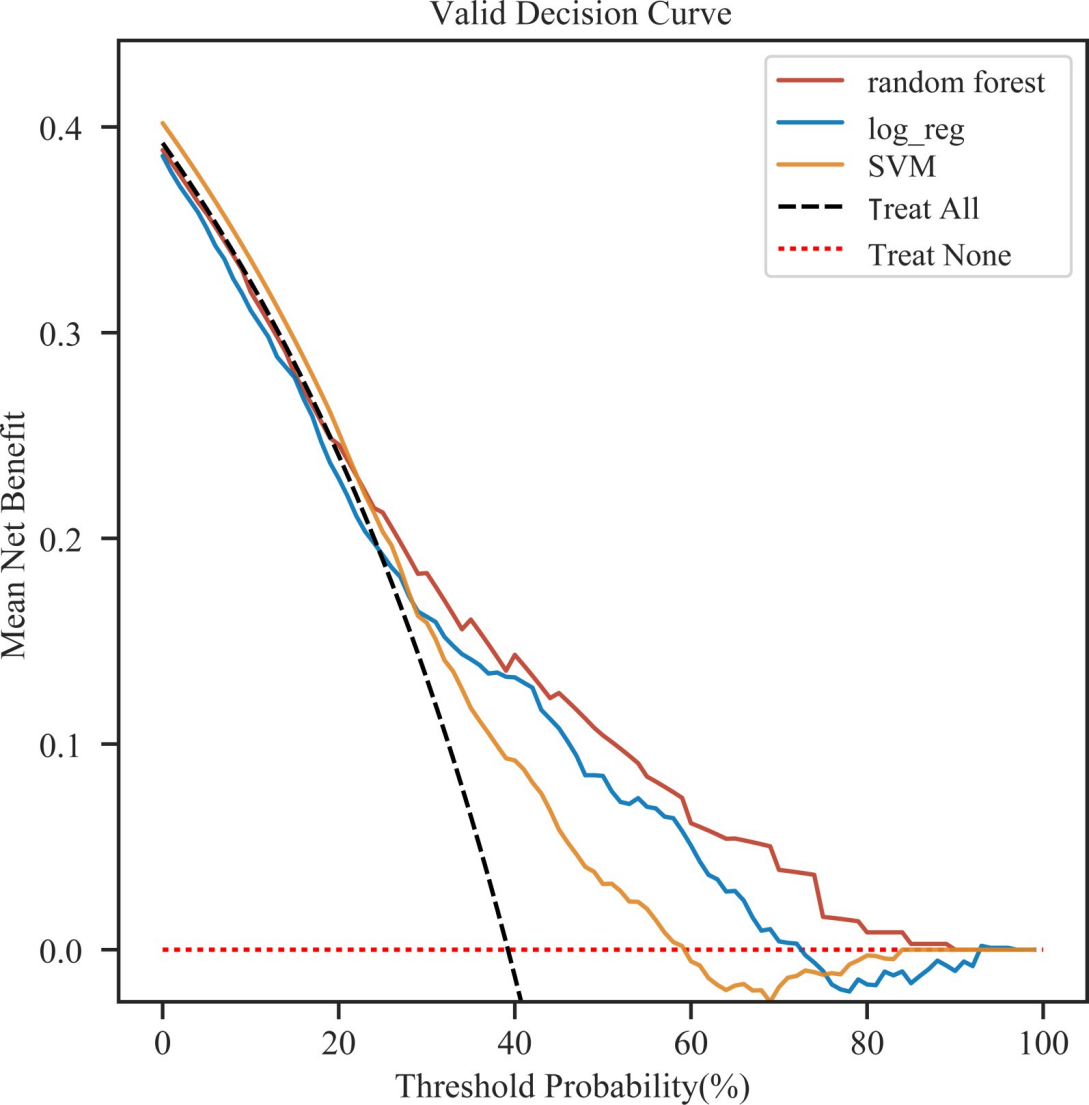
DCA curves of the top three best-performing models.

**Fig 5:**
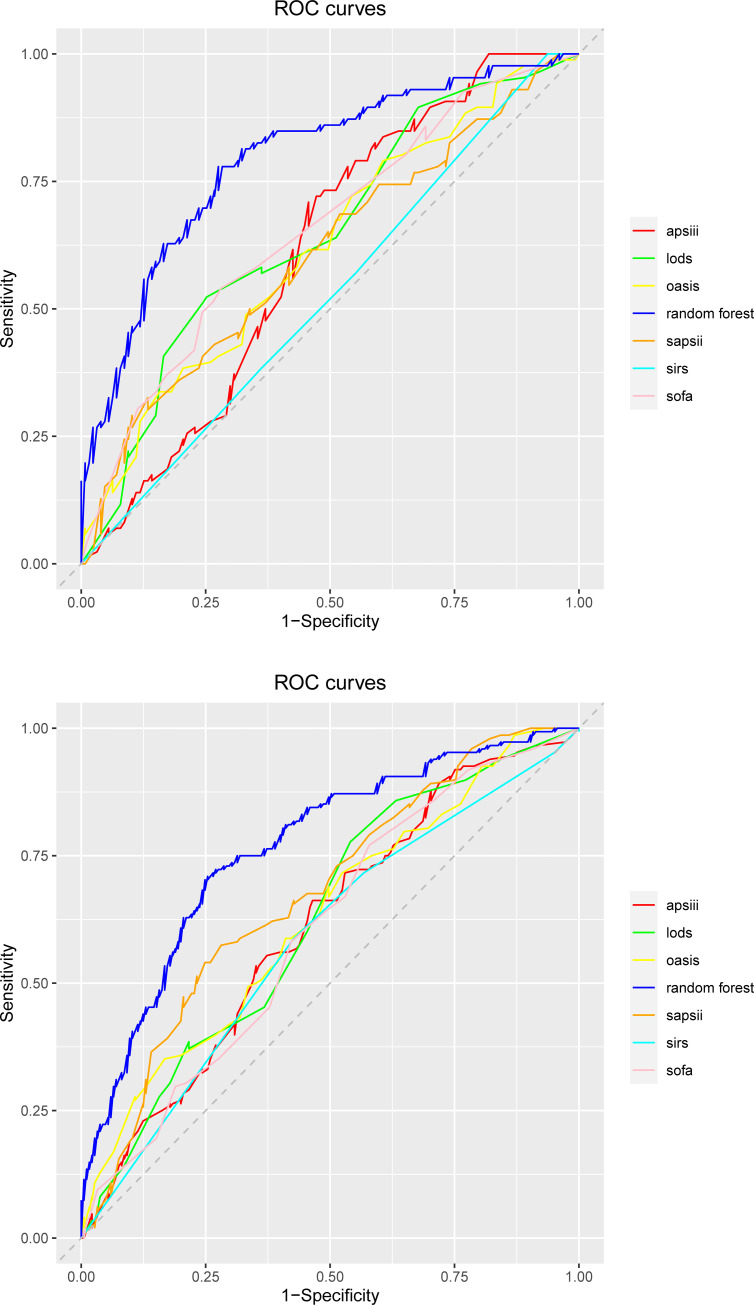
ROC curves of the random forest and traditional disease severity scores. **A.** Internal validation set. **B.** External validation set.

We selected the top three models in terms of AUC value for Decision Curve Analysis (DCA), and RF remained the best performing model among them (Fig 4). The RF model showed better predictive performance when compared to the traditional disease severity scores (SIRS (AUC.0.53 (95% CI: 0.45–0.60)), SOFA (AUC. 0.62 (95% CI: 0.55–0.70)), OASIS (AUC. 0.62 (95% CI: 0.55–0.70)), SAPSII (AUC. 0.61 (95% CI: 0.53–0.69)), LODS (AUC. 0.65 (95% CI: 0.58–0.73)), and APSIII (AUC. 0.61 (95% CI: 0.54–0.69)). The ROCs are shown in Fig 5, and Table 3 compared performance evaluation of RF and traditional disease severity score in the internal validation set. RF was the optimal model for predicting 28-day mortality in patients with SALI. We also compared the predictive performance of the models in the external validation set and the results are shown in S6 and S7 Tables. Hyperparameter tuning resulted in better predictive performance of the model, and Table 4 displays the result.

**Table 3:** Compared performance evaluation of random forest and traditional disease severity scores in predicting 28-day mortality rate in the internal validation set.

Model/scores	AUC	Accuracy(%)	Precision(%)		Recall	Specificity(%)	
**Random forest**	0.79	74.65	75.52	0.85			59.30
**Apsiii**	0.61	55.40	61.11	0.69			34.89
**Lods**	0.65	62.44	63.91	0.85			29.07
**Oasis**	0.62	63.83	65.24	0.84			33.72
**Sapsii**	0.61	58.69	64.44	0.69			44.19
**Sirs**	0.53	59.62	59.62	1.00			0.00
**Sofa**	0.62	64.32	64.25	0.91			25.58

**Table 4:** Compared performance of the random forest model before and after hyperparameter tuning.

Random Forest (parameter set)	AUC	Acc(%)	Precision(%)		Recall	Specificity(%)
**Before**	0.79	74.65	75.52	0.85		59.30
**After**	0.80	74.65	75.52	0.85		59.30

### Model explainability

**Fig 6:**
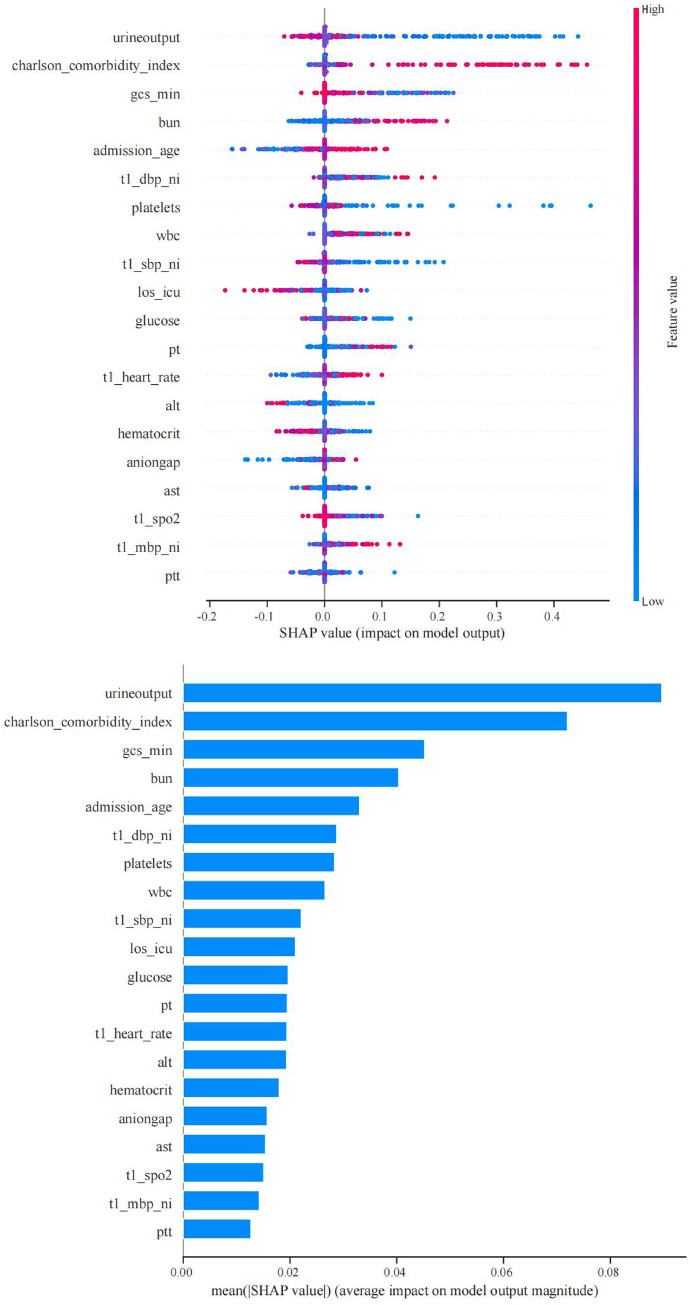
SHAP summary chart. **A.** SHAP values showing the influence of different features on the output of RF Model. **B.** Mean absolute SHAP values for each clinical feature.

To improve the clinical utility of the model, we used the SHAP method to determine which features contribute to the model’s prediction of 28-day mortality in patients with sepsis, which is shown in Fig 6. Fig 6A shows the distribution of the SHAP values of the top 20 clinical features: each point in the figure represents a feature, and the position of the point indicates the SHAP value of the feature, with the value representing the magnitude of the feature’s contribution to the model output. If the value is positive, the feature positively influences the output; if the value is negative, the feature negatively influences the output. Red color indicates high values and blue color indicates low values. A darker color indicates a stronger influence of the feature on the target feature. Fig 6A shows a low SHAP value for urine output and GCS_min that indicated a positive influence on 28-day mortality, while the CCI, BUN and admission_age displayed an opposite trend. The bar chart was formed by ranking the features from high to low according to their average SHAP absolute values, indicating the degree of the contribution of each feature to the whole model. The larger the SHAP absolute value is, the more important the feature is, and the greater impact it has on the model output results. From Fig 6B, it is easy to see that the top five clinically important features were urin output, CCI, GCS_min, BUN and admission_age.

**Fig 7:**
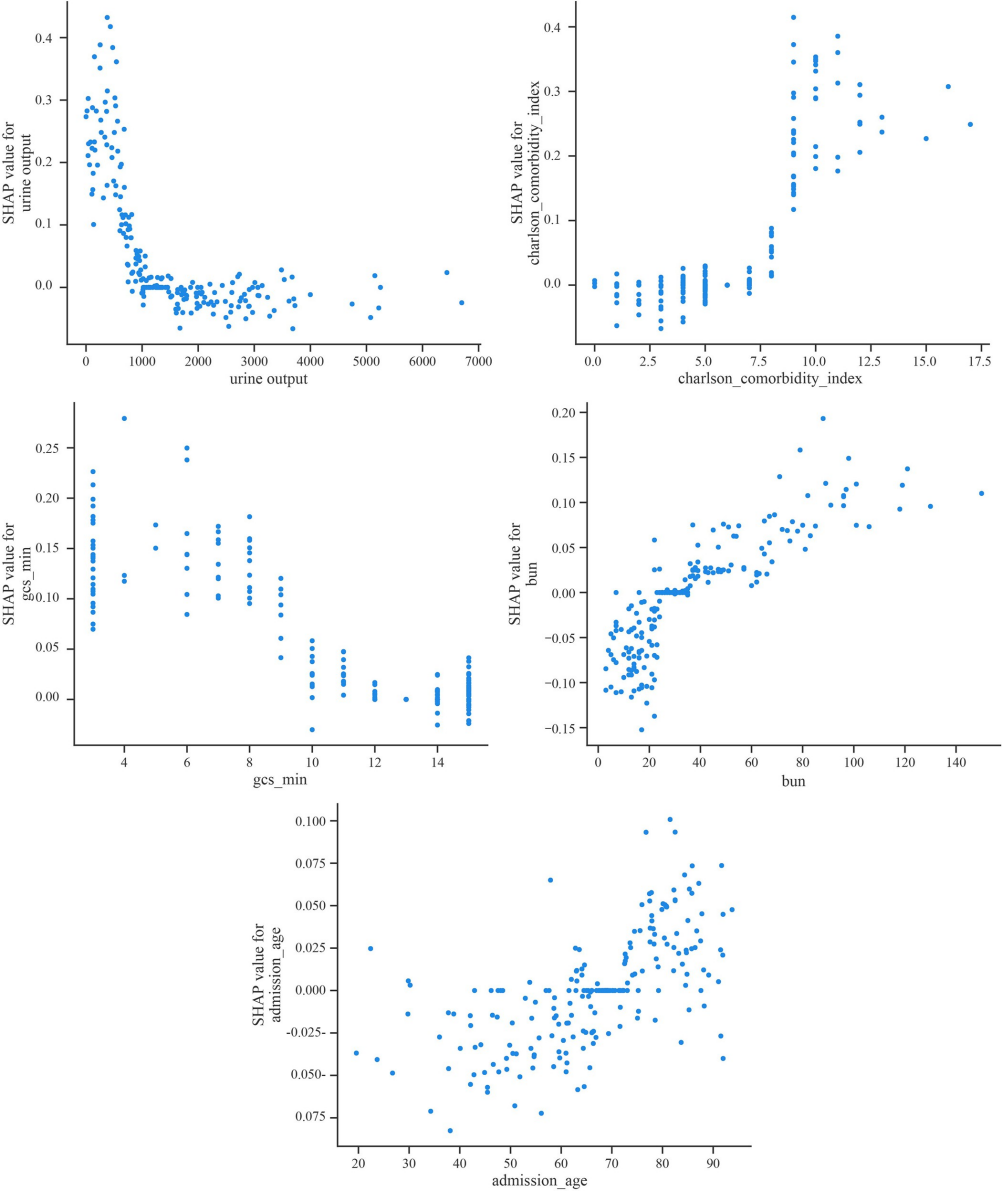
SHAP dependency plot of the top 5 influential clinical features on model outcomes. **A.** Urine output; **B.** CCI; **C.** GCS_min; **D.** BUN; **E.** Admission_age.

Based on the summary plot of SHAP, we further derived the top 5 influential SHAP dependency plots to explain the effect of clinical characteristics on the risk of 28-day death (Fig 7). The vertical axis of the SHAP dependency plot is the SHAP value of the clinical characteristic, while the horizontal axis is the range of variation of the clinical characteristic, where a SHAP value higher than zero indicates that the patient has an increased risk of 28-day death.

## Discussion

We developed and validated a predictive model using a large dataset in order to build a valid, stable, and interpretable model to predict 28-day mortality in patients with SALI. Among the multiple models developed, RF was the most reliable and stable and had the best predictive performance. We compared RF with the traditional disease severity scores (SIRS, SOFA, OASIS, SAPSII, LODS, and APSIII) and found that the RF model was still performed the best. An external validation of the model was performed, confirming the stability of RF. To date, no researchers have developed a predictive model for 28-day mortality in patients with SALI, and no studies have used multi-model screening for optimal model development. Some researchers have used nomogram to predict in-hospital mortality and 90-day mortality in patients with SALI, but they have only compared the developed model with some of the traditional disease severity scores [22,23]. We also performed hyperparameter tuning of the model after developing the optimal model to optimize the predictive performance of the model [24,25]. In addition, we screened the five clinical features that contributed most to the model, which were Urine output, CCI, GCS, BUN, and admission_age. Therefore, clinical features can serve as early warning.

Shapley value was used to explain the opacity of the model [26]. Model opacity refers to the opacity of the intermediate process between the input of data and the output of results [27,28]. From Fig 7A, Shapley value was 0 when the urine volume was about 1000 ml within 24 h after admission, and the Shapley value decreased, which showed a negative effect on 28-d mortality in SALI, when the urine volume increased. The GCS value for a Shapley value of 0 is approximately 10 from Fig 7C, and its effect on 28-d mortality in SALI is consistent with urine output. However, the effect of CCI, BUN and admission_age on 28-day mortality in SALI were opposite to the trend of the first two features. The Shapley values tended to approach 0 when the CCI, BUN and admission_age were about 6, 22 and 65, respectively.

It is well known that patients with severe sepsis have severely impaired microcirculation and reduced end-organ tissue perfusion, exacerbating organ damage. Urine output is one of the traditional indicators of tissue perfusion that can be used to assess microcirculation [29]. A study by Heffernan et al. on the relationship between urine output and mortality in critically ill patients showed that a urine output threshold of less than 0.5 mL/kg/hr moderately predicted mortality in ICU inpatients [30]. This serves as a reminder to clinicians that they need to focus not only on the total amount of urine output, but also on changes to urine output over time to detect changes in the patient’s microcirculatory concerns in a timely manner. Bun is one of the indicators used to assess kidney function. wen et al. showed that bun greater than or equal to 21 mg/dl is one of the most important predictors of mortality risk in patients with sepsis, which is almost consistent with our results [31]. The CCI, developed in 1987, is considered the gold standard for assessing comorbidities in clinical studies [32], as a tool used to predict long-term mortality in patients [33]. Previous studies have also shown an increase in patient mortality with increasing CCI [34,35], consistent with our results. We usually use the GCS which is a scale used to assess a patient’s level of consciousness [36]. Lai Q et al. incorporated GCS into a model construct to assess in-hospital mortality in patients with sepsis. Our model and that of Lai Q et al. consistently show that GCS is an important clinical indicator in predicting the risk of death in patients with sepsis [37]. As for the admission_age, as people aging, their bodily functions gradually deteriorate, and the functioning of their organs diminishes. This may explain why admission_age was one of the top five important predictors of 28-day mortality in patients with sepsis-related liver injury.

We found that the top 5 metrics that had the greatest impact on predicting performance were not liver function-related metrics. The ALBI grade is a new score for assessing liver function, which was developed by Dr. Philip J. Johnson, Professor of Translational Oncology at the University of Liverpool, UK [38]. However, due to too many missing values, more than 40% (S4 Table), it was excluded when incorporating the clinical features used to construct the model, and in Table 1, no statistically significant difference between the two groups of ABLI in the SALI death group and survival group. Moreover, liver function related measurements, except ALP death group was significantly higher than the stock group, other measurements of the two groups of patients were not significantly different, in Table 1. SALI is a hepatic impairment caused by sepsis, usually accompanied by other organ injuries, only liver function impairment-related indexes are not sufficient to represent the overall severity of this group of patients, and there is no significant difference between liver function-related indexes in the death group and the survival group, they may be the reason for the absence of indicators of liver injury among the five most important indicators affecting the predictive performance of the model.

There are some limitations in our study. First, our modeling used a single-center dataset and was a retrospective study; In addition, non-overlapping dataset with MIMIC-IV in MIMIC-III was used as an external validation queue, and the chronology was not forward-looking; Third, we focused only on the clinical indicators within 24 h after ICU admission and did not assess the impact of changes in the clinical features on the outcomes during the ICU stay. Therefore, further design of multicenter prospective studies is needed to validate our findings.

## Conclusion

RF machine learning models have good predictive ability for 28-day mortality prediction in SALI. Urine output, CCI, GCS-min, BUN and admission age within 24 h of ICU admission contribute significantly to model prediction.

## Supporting information

S1 Fig Distribution of the original and interpolated data. (DOCX)

S1 Table All extracted variables collection from the MIMIC-IV and MIMIC-III database. (CSV)

S2 Table TRIPOD checklist. (CSV)

S3 Table Missing number (%) for included variables in the dataset (MIMIC-IV). (CSV)

S4 Table A list of the features that were finally used for prediction in this study. (CSV)

S5 Table Baseline characteristics of the cohort from MIMIC-III. (CSV)

S6 Table Compare the performance evaluation of 8 machine learning classific-ation models in predicting 28-day mortality rate in the external validation set. (CSV)

S7 Table Compare performance evaluation of random forest and traditional disease severity scores in predicting 28-day mortality rate in the external validation set. (CSV)

S1 File (DOCX)
