# Supplementary figures and images for: An interpretable machine learning model for predicting 28-day mortality in patients with sepsis-associated liver injury
Source: PLoS One. 2024 May 20;19(5):e0303469. doi: 10.1371/journal.pone.0303469 (PMC11104601; doi:10.1371/journal.pone.0303469)

**S1 Fig. Distribution of the original and interpolated data.**


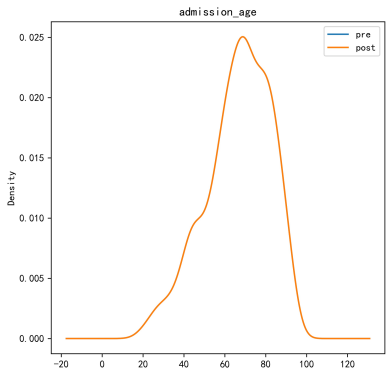

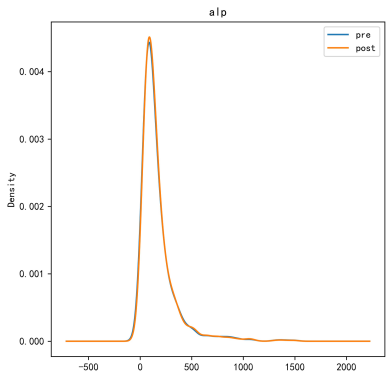

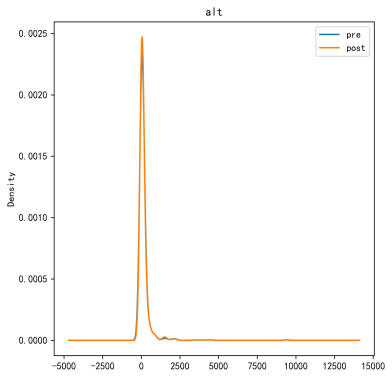

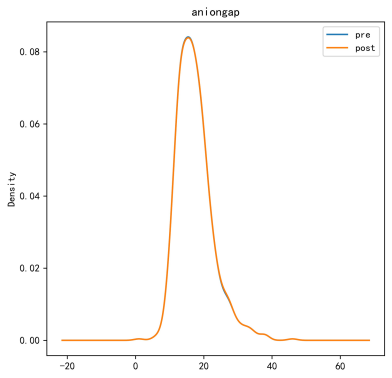

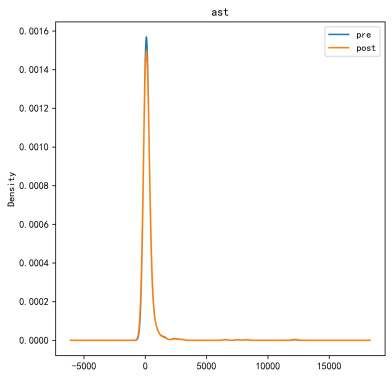

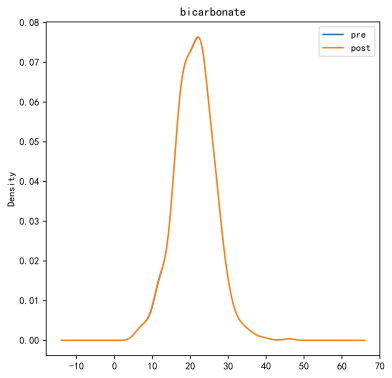

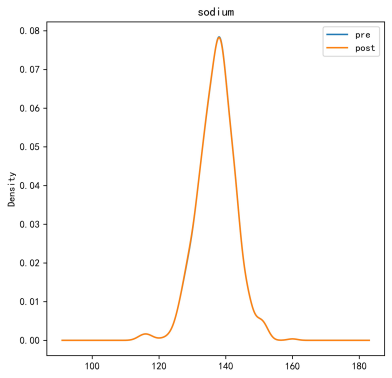

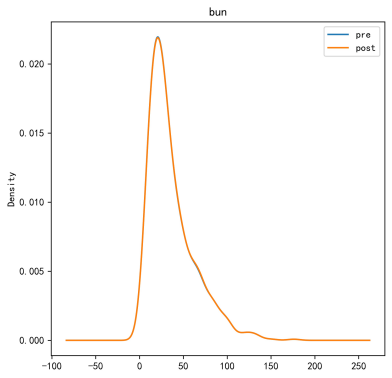

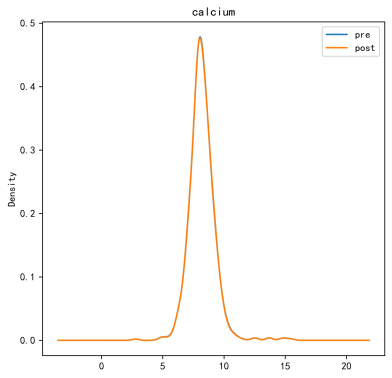

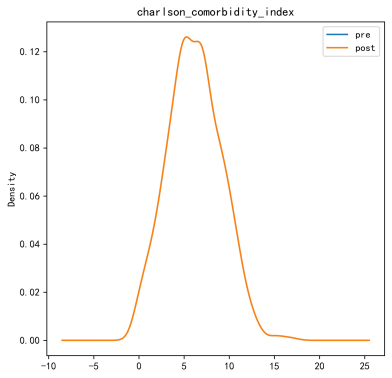

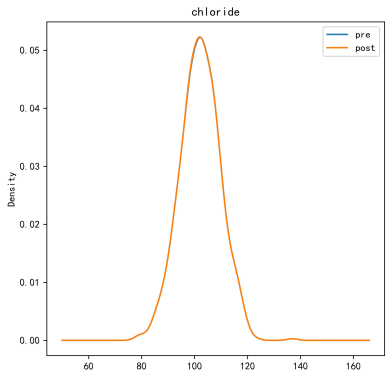

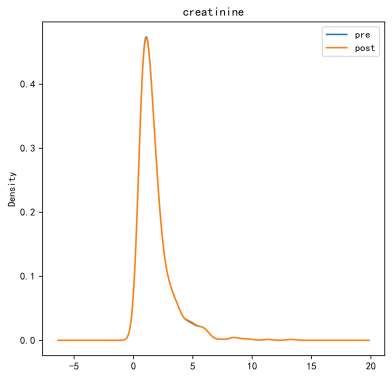

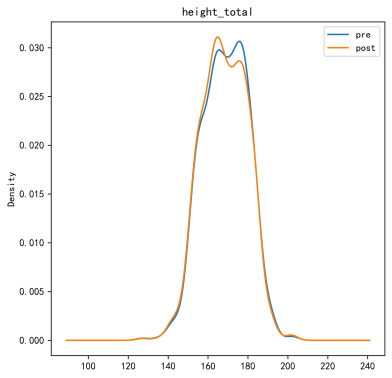

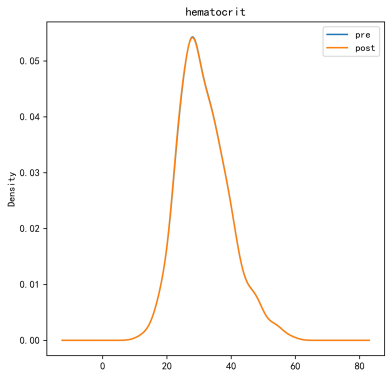

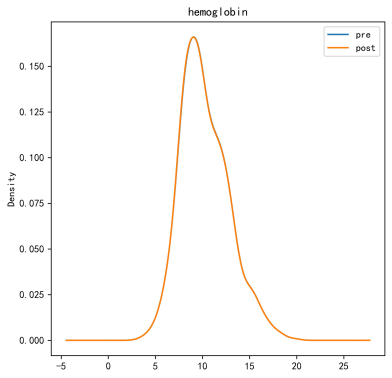

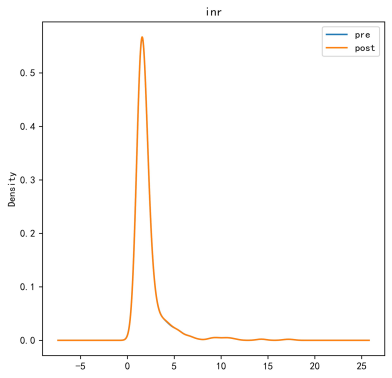

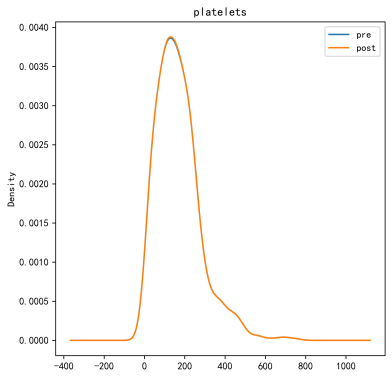

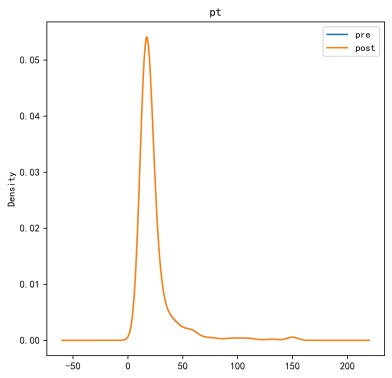

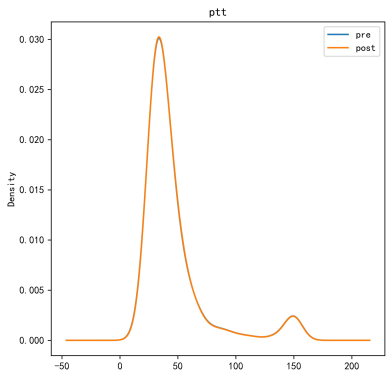

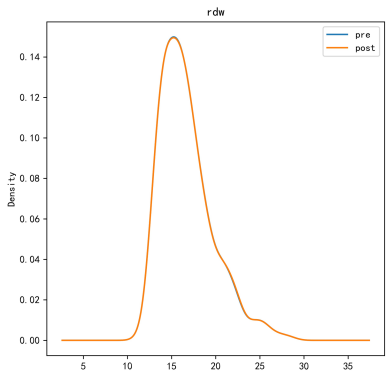

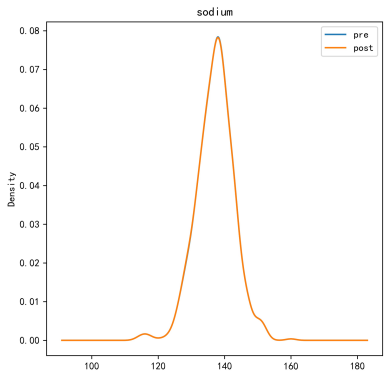

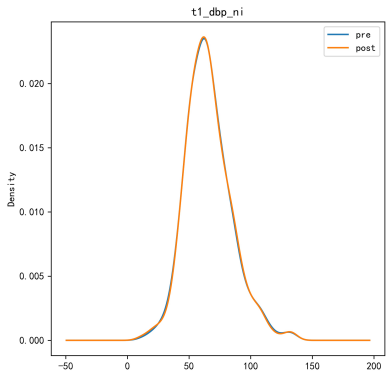

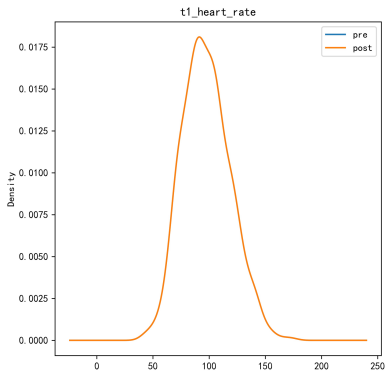

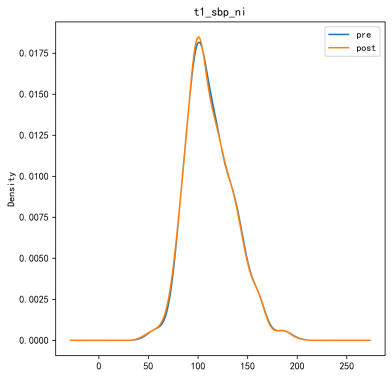

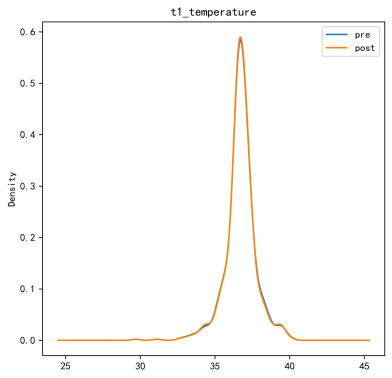

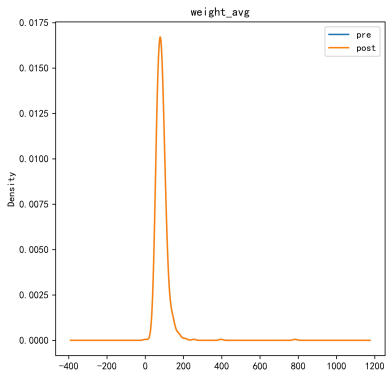

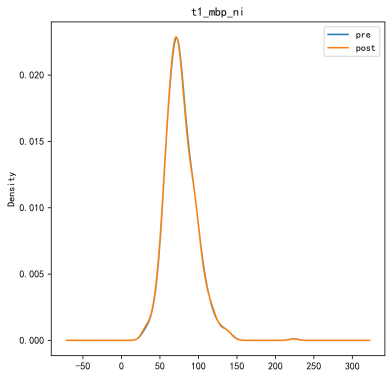

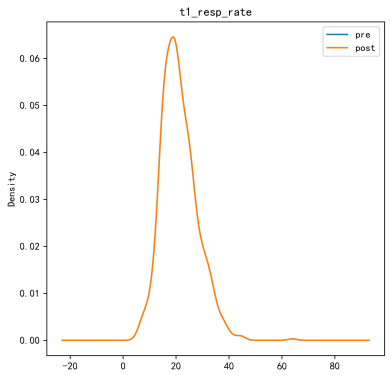

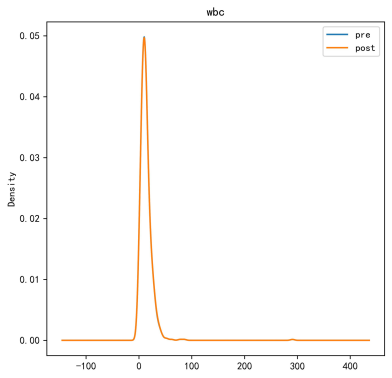

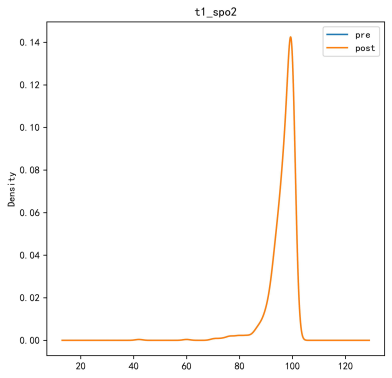

Supplement: S1 Fig — (DOCX) [file pone.0303469.s001.docx]
