# Supplementary material for: An interpretable machine learning model for predicting 28-day mortality in patients with sepsis-associated liver injury
Source: PLoS One. 2024 May 20;19(5):e0303469. doi: 10.1371/journal.pone.0303469 (PMC11104601; doi:10.1371/journal.pone.0303469)
Supplement: S1 File — (DOCX) [file pone.0303469.s009.docx]

**S1 Table. All extracted variables collection from the MIMIC-IV and MIMIC-III database.**

| Age | **Demographic ch aracteristics (5)** |
| --- | --- |
| Sex |  |
| Body weight |  |
| Height |  |
| BMI |  |
| Charlson Comorbidity Index | **Medical history (9)** |
| Hypertension |  |
| Diabetes |  |
| Congestive heart failure |  |
| Myocardial infarction |  |
| Peptic ulcer |  |
| Cerebrovascular disease |  |
| Chronic pulmonary disease |  |
| Renal disease |  |
| Heart rate | **Vital signs (7)** |
| Systolic blood pressure |  |
| Diastolic blood pressure |  |
| Mean artery pressure |  |
| Respiratory rate |  |
| Body temperature |  |
| SpO_2_ |  |
| White blood cell | **Laboratory parameters(44)**  **（Data from the first laboratory examination after admission to the ICU）** |
| Neutrophil |  |
| Lymphocyte |  |
| Red blood cell distribution width |  |
| Platelets |  |
| Hematocrit |  |
| Hemoglobin |  |
| Hypersensitive c-reactive protein |  |
| D_dimer |  |
| Prothrombin Time |  |
| International normalized ratio |  |
| Partial thromboplastin time |  |
| Fibrinogen |  |
| Alanine aminotransferase |  |
| Alkaline phosphatase |  |
| Aspartate aminotransferase |  |
| Amylase |  |
| Total bilirubin |  |
| Lactate dehydrogenase |  |
| Albumin |  |
| ABLI |  |
| Triglyceride |  |
| High-density lipoprotein |  |
| Low-density lipoprotein |  |
| Blood urea nitrogen |  |
| Serum creatinine |  |
| Creatine phosphokinase |  |
| High sensitivity troponin_i |  |
| Creatine kinase MB |  |
| High sensitivity troponin T |  |
| N-terminal pro brain natriuretic peptide |  |
| Lactate |  |
| pH |  |
| pO_2_ |  |
| pCO2 |  |
| PaO_2_/FiO_2_ ratio |  |
| Base excess |  |
| Anion gap |  |
| Bicarbonate |  |
| Serum calcium |  |
| Serum chloride |  |
| Serum sodium |  |
| Serum potassium |  |
| Blood glucose |  |
| Duration of ICU stay this time | **Others (6)** |
| Site of infection |  |
| Dopamine(ug/kg.min) |  |
| Adrenalin(ug/kg.min) |  |
| Noradrenaline(ug/kg.min) |  |
| Dobutamine(ug/kg.min) |  |
| Urine output on day 1 | **Other(1)** |
| GCS | **Scoring to assess disease severity（7）** |
| SIRS |  |
| SOFA |  |
| OASIS |  |
| SAPSII |  |
| LODS |  |
| APSIII |  |
| 28-day mortality | **Outcome (1)** |

**S2 Table.** **TRIPOD checklis.**

| **Section/Topic** | **Item** | **Checklist Item** | **Page** |
| --- | --- | --- | --- |
| **Title and abstract** | | | |
| Title | 1 | Identify the study as developing and/or validating a multivariable prediction model, the target population, and the outcome to be predicted. | **1** |
| Abstract | 2 | Provide a summary of objectives, study design, setting, participants, sample size, predictors, outcome, statistical analysis, results, and conclusions. | **1-2** |
| **Introduction** | | | |
| Background and objectives | 3a | Explain the medical context (including whether diagnostic or prognostic) and rationale for developing or validating the multivariable prediction model, including references to existing models. | **2-4** |
|  | 3b | Specify the objectives, including whether the study describes the development or validation of the model or both. | **4** |
| **Methods** | | | |
| Source of data | 4a | Describe the study design or source of data (e.g., randomized trial, cohort, or registry data), separately for the development and validation data sets, if applicable. | **4** |
|  | 4b | Specify the key study dates, including start of accrual; end of accrual; and, if applicable, end of follow-up. | **4** |
| Participants | 5a | Specify key elements of the study setting (e.g., primary care, secondary care, general population) including number and location of centres. | **4** |
|  | 5b | Describe eligibility criteria for participants. | **4** |
|  | 5c | Give details of treatments received, if relevant. | **no** |
| Outcome | 6a | Clearly define the outcome that is predicted by the prediction model, including how and when assessed. | **6-7** |
|  | 6b | Report any actions to blind assessment of the outcome to be predicted. | **6-7** |
| Predictors | 7a | Clearly define all predictors used in developing or validating the multivariable prediction model, including how and when they were measured. | **4-5** |
|  | 7b | Report any actions to blind assessment of predictors for the outcome and other predictors. |  |
| Sample size | 8 | Explain how the study size was arrived at. | **8** |
| Missing data | 9 | Describe how missing data were handled (e.g., complete-case analysis, single imputation, multiple imputation) with details of any imputation method. | **6** |
| Statistical analysis methods | 10a | Describe how predictors were handled in the analyses. | **6** |
|  | 10b | Specify type of model, all model-building procedures (including any predictor selection), and method for internal validation. | **6-7** |
|  | 10d | Specify all measures used to assess model performance and, if relevant, to compare multiple models. | **6-7** |
| Risk groups | 11 | Provide details on how risk groups were created, if done. |  |
| **Results** | | | |
| Participants | 13a | Describe the flow of participants through the study, including the number of participants with and without the outcome and, if applicable, a summary of the follow-up time. A diagram may be helpful. | **8-9** |
|  | 13b | Describe the characteristics of the participants (basic demographics, clinical features, available predictors), including the number of participants with missing data for predictors and outcome. | **8-12** |
| Model development | 14a | Specify the number of participants and outcome events in each analysis. | **8-9** |
|  | 14b | If done, report the unadjusted association between each candidate predictor and outcome. | **8-12** |
| Model specification | 15a | Present the full prediction model to allow predictions for individuals (i.e., all regression coefficients, and model intercept or baseline survival at a given time point). | **17-18** |
|  | 15b | Explain how to the use the prediction model. | **18-19** |
| Model performance | 16 | Report performance measures (with CIs) for the prediction model. | **13** |
| **Discussion** | | | |
| Limitations | 18 | Discuss any limitations of the study (such as nonrepresentative sample, few events per predictor, missing data). | **22** |
| Interpretation | 19b | Give an overall interpretation of the results, considering objectives, limitations, and results from similar studies, and other relevant evidence. | **20-22** |
| Implications | 20 | Discuss the potential clinical use of the model and implications for future research. | **20** |
| **Other information** | | | |
| Supplementary information | 21 | Provide information about the availability of supplementary resources, such as study protocol, Web calculator, and data sets. | **23** |
| Funding | 22 | Give the source of funding and the role of the funders for the present study. | **22** |

**S3 Table. Missing number (%) for included variables in the dataset(MIMIC-IV).**

| **Characteristics** | **Missing, N (%)** |
| --- | --- |
| **Demographic** |  |
| Age, year | 0(0) |
| Sex | 0(0) |
| Male, n (%) |  |
| Female, n (%) |  |
| Weight, kg | 9(1.3) |
| Height, cm  BMI | 91(12.8)  288(40.6) |
| **Comorbidities** |  |
| Charlson Comorbidity Index | 0(0) |
| Hypertension,n(%) | 0(0) |
| Diabetes,n(%) | 0(0) |
| Congestive heart failure ,n(%) | 0(0) |
| Myocardial infarction,n(%) | 0(0) |
| Peptic ulcer,n(%) | 0(0) |
| Cerebrovascular disease,n(%) | 0(0) |
| Chronic pulmonary disease,n(%) | 0(0) |
| Renal disease,n(%) | 0(0) |
| **Vital signs on day 1** |  |
| Heart rate, bpm | 0(0) |
| Systolic blood pressure, mmHg | 95(13.4) |
| Diastolic blood pressure, mmHg | 95(13.4) |
| Mean arterial pressure, mmHg | 95(13.4) |
| Respiratory rate | 0(0) |
| Body temperature, ℃ | 53(7.5) |
| SpO_2_, % | 0(0) |
| **Laboratory findings on day 1** |  |
| White blood cell, ×10^3^/uL | 3(0.4) |
| Neutrophils,%  Neutrophils,%  Red blood cell distribution width | 197(27.7)  197(27.7)  3(0.4) |
| Platelets, ×10^3^/uL | 2(0.3) |
| Hematocrit, % | 2(0.3) |
| Hemoglobin, g/dL  Hypersensitive c-reactive protein,mg/L  Prothrombin Time,s | 2(0.3)  691(97.3)  9(1.3) |
| D_dimer,ng/mL | 703(99) |
| International normalized ratio  Partial thromboplastin time,s  Fibrinogen，mg/dL | 9(1.3)  12(1.7)  350(49.3) |
| Alanine aminotransferase, U/L | 100(14.1) |
| Alkaline phosphatase, U/L | 102(14.4) |
| Aspartate aminotransferase, U/L | 98(13.8) |
| Amylase, U/L | 577(81.3) |
| Total bilirubin, mg/dL  Lactate dehydrogenase，U/L | 95(13.4)  279(39.3) |
| Albumin, g/L | 247(34.8) |
| ABLI | 301(42.4) |
| Triglyceride，mg/dL  High-density lipoprotein，mg/dL  Low-density lipoprotein，mg/dL | 666(93.8)  697(98.2)  698(98.3) |
| Blood urea nitrogen, mg/dL | 2(0.3) |
| Creatine phosphokinase,U/L | 429(60.4) |
| Serum creatinine, mg/dL  Creatine kinase MB, U/L  High sensitivity troponin T,ug/L  N-terminal pro brain natriuretic peptide,pg/mL | 2(0.3)  423(59.6)  449(63.2)  613(86.3) |
| Lactate, mmol/L | 245(34.5) |
| pH | 215(30.3) |
| pO_2_, mmHg | 215(30.3) |
| pCO_2_, mmHg | 215(30.3) |
| PaO_2_/FiO_2_ ratio | 304(42.8) |
| Base excess | 215(30.3) |
| Anion gap | 2(0.3) |
| Bicarbonate, mmol/L | 2(0.3) |
| Serum calcium, mmol/L | 2(0.3) |
| Serum chloride, mmol/L | 2(0.3) |
| Serum sodium, mmol/L | 2(0.3) |
| Serum potassium, mmol/L | 2(0.3) |
| Blood glucose, mg/dL | 5(0.7) |
| **Duration of ICU stay this time , day**  Site of infection  Dopamine(ug/kg.min)  Adrenalin(ug/kg.min)  Noradrenaline(ug/kg.min)  Dobutamine(ug/kg.min) | 0(0)  0(0)  668(94.1)  601(84.6)  333(46.9)  666(93.8) |
| **Urine output on day 1, mL** | 30(4.2) |
| **Severity of illness scores** |  |
| GCS  SIRS  SOFA  OASIS | 0(0)  0(0)  0(0)  0(0) |
| SAPSII | 0(0) |
| LODS  APSIII  28-day mortality | 0(0)  0(0)  0(0) |

**S1 Figure. Distribution of the original and interpolated data.**


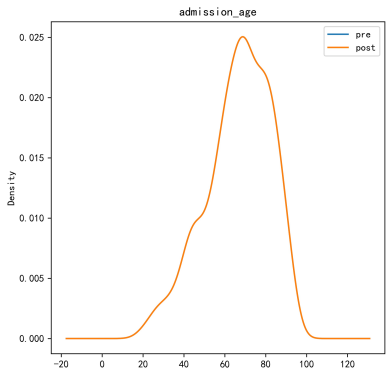

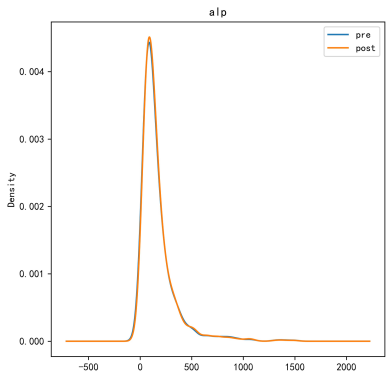

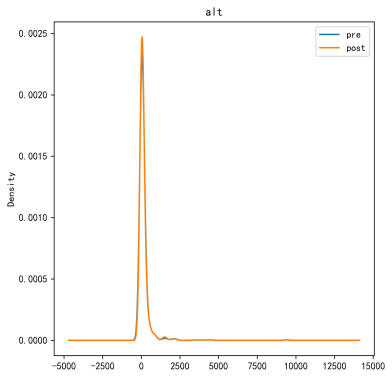

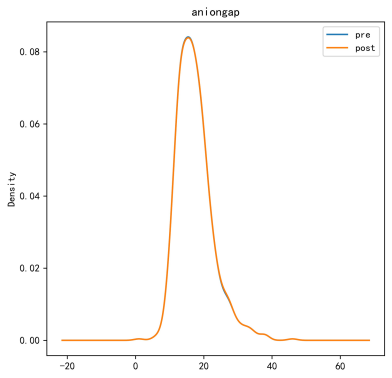

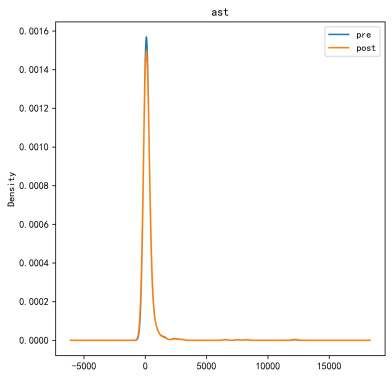

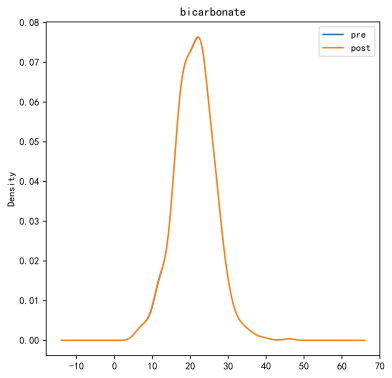

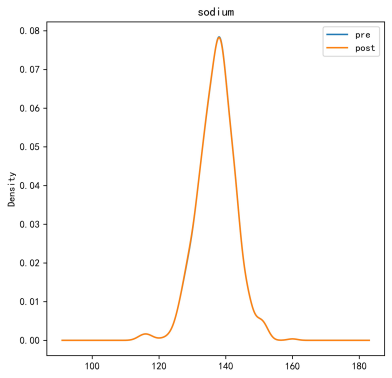

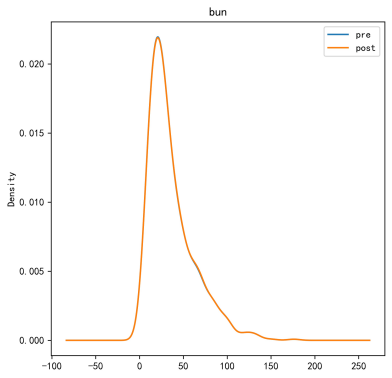

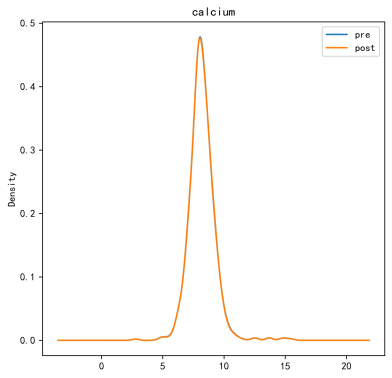

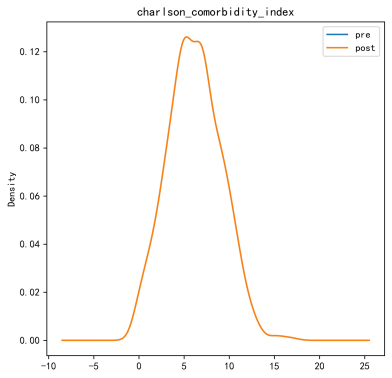

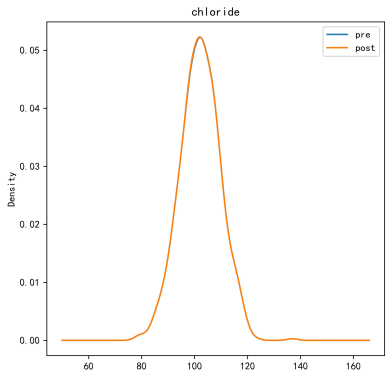

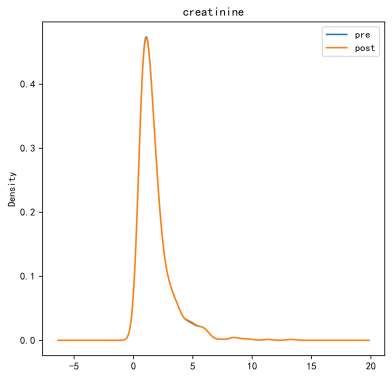

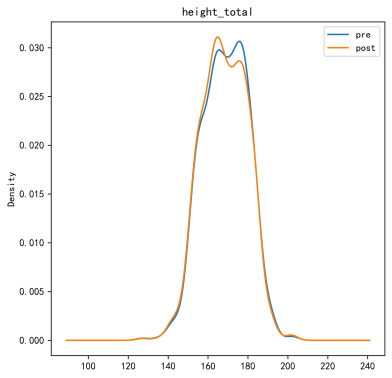

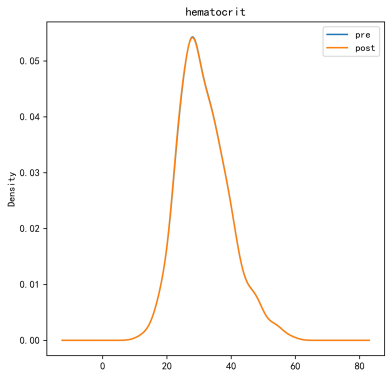

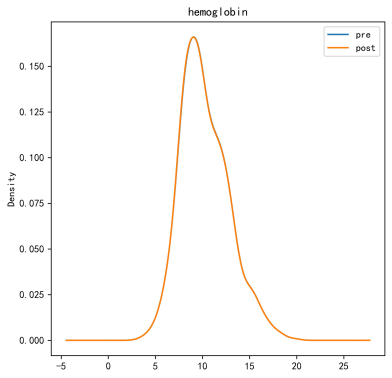

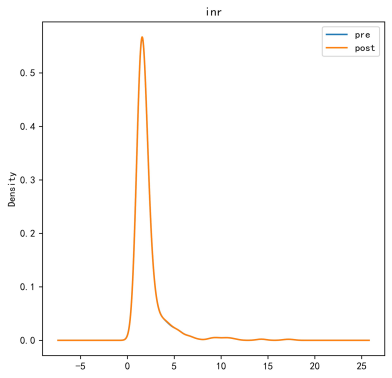

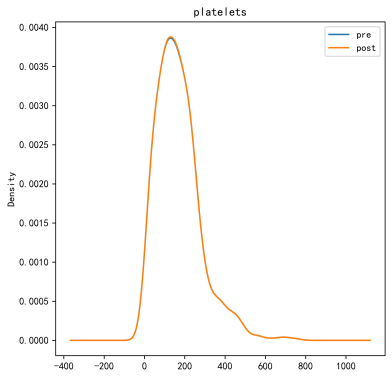

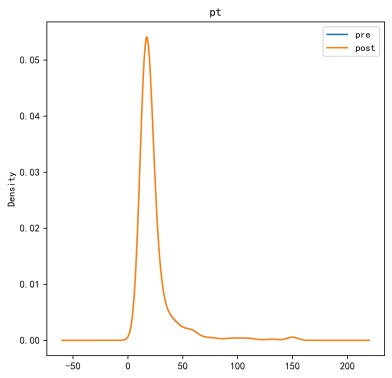

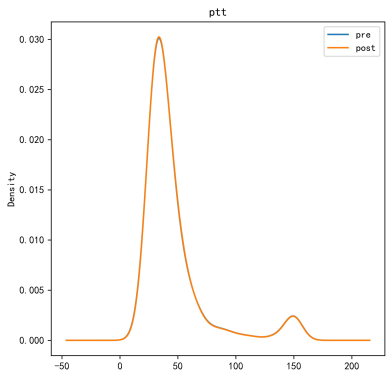

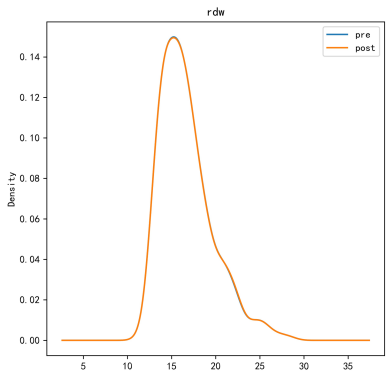

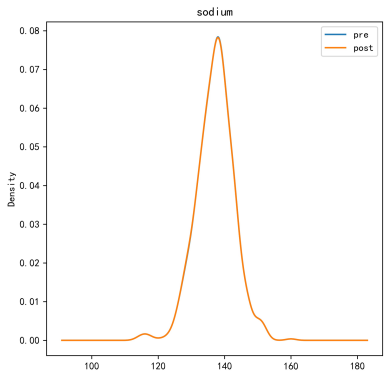

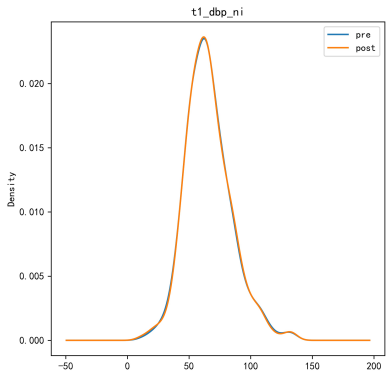

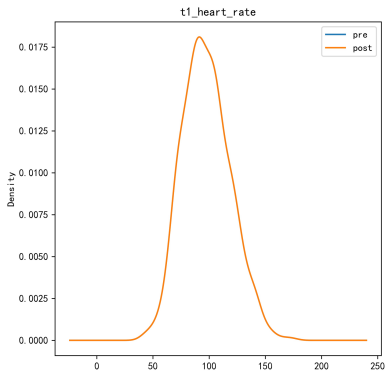

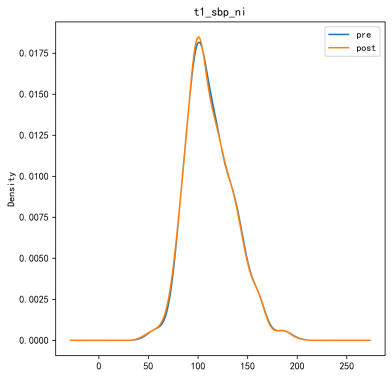

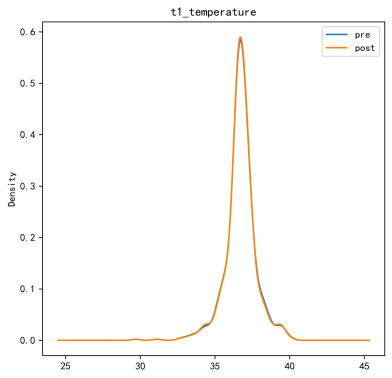

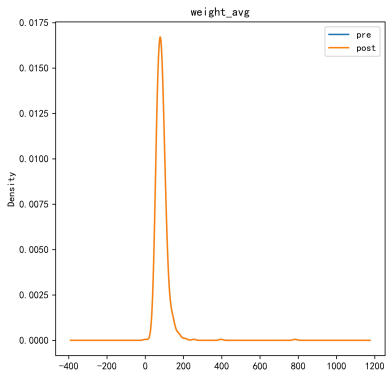

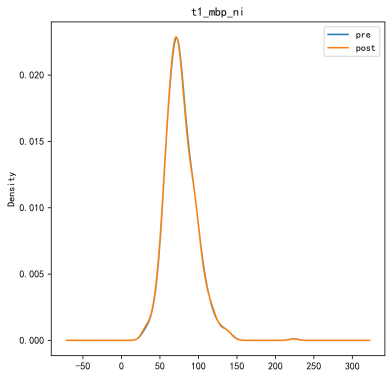

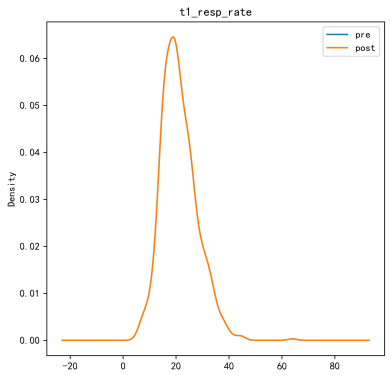

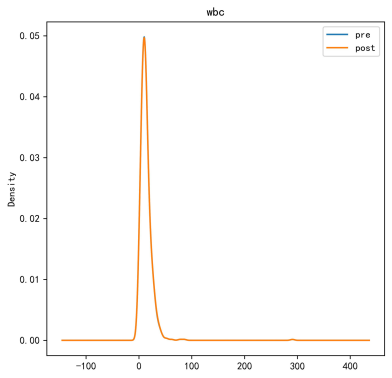

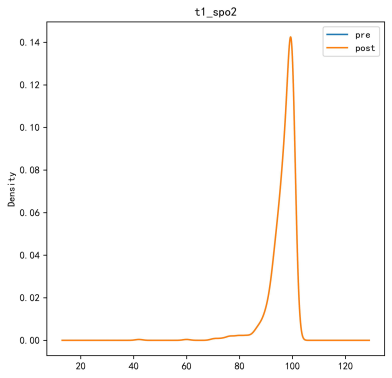


**S4 Table.** **A list of the features that were finally used for prediction in this study.**

| Age | **Demographic characteristics (2)** |
| --- | --- |
| Height |  |
| Charlson Comorbidity Index | **Medical history (1)** |
| Heart rate | **Vital signs (6)** |
| Systolic blood pressure |  |
| Diastolic blood pressure |  |
| Mean artery pressure |  |
| Respiratory rate |  |
| SpO_2_ |  |
| White blood cell | **Laboratory parameters(12)**  **（Data from the first laboratory examination after admission to the ICU）** |
| Platelets |  |
| Hematocrit |  |
| Prothrombin Time |  |
| Partial thromboplastin time |  |
| Alanine aminotransferase |  |
| Alkaline phosphatase |  |
| Aspartate aminotransferase |  |
| Blood urea nitrogen |  |
| Anion gap |  |
| Serum chloride |  |
| Blood glucose |  |
| Duration of ICU stay this time | **Others (1)** |
| Urine output on day 1 | **Urine (1)** |
| GCS | **Severity of illness scores(1)** |

**S5 Table . Baseline characteristics of the cohort from MIMIC-III**

| **Characteristics** | **All（N=333）** | **Survival(N=185)** | **Non-survival(N=148)** | **P value** |
| --- | --- | --- | --- | --- |
| **Demographic** |  |  |  |  |
| Age, year | 67.6(55.7,77.8) | 65.6(48.6,77.2) | 70.1(61.1,78.8) | 0.002 |
| Sex |  |  |  | 0.764 |
| Male, n (%) | 201(60.3) | 113(61.1) | 88(59.5) |  |
| Female, n (%) | 132(39.6) | 72(38.9) | 60(40.5) |  |
| Weight, kg | 76.8(64.9,90) | 77.3(65.4,89.4) | 74.7(63.3,92.9) | 0.898 |
| Height, cm | 170.2(162.6,177.8) | 170.2(163.8,177.8) | 172.7(162.6,177.8) | 0.427 |
| BMI | 27.1(23.1,30.2) | 27.00(23.00,29.86) | 27.31(23.20,31.98) | 0.343 |
| **Comorbidities** |  |  |  |  |
| Charlson Comorbidity Index | 6(4,7) | 5(3,7) | 6(5,8) | <0.001 |
| Diabetes,n (%) | 66(19.8) | 30(16.2) | 36(24.3) | 0.065 |
| Congestive heart failure,n (%) | 161(48.3) | 83(44.9) | 78(52.7) | 0.155 |
| Myocardial infarction,n (%) | 57(17.1) | 30(16.2) | 27(18.2) | 0.626 |
| Peptic ulcer,n (%) | 7(2.1) | 4(2.2) | 3(2.0) | 0.932 |
| Cerebrovascular disease,n (%) | 20(6.0) | 11(5.9) | 9(6.1) | 0.959 |
| Chronic pulmonary disease,n (%) | 60(18.0) | 33(17.8) | 27(18.2) | 0.924 |
| Renal disease,n (%) | 56(16.8) | 26(14.1) | 30(20.3) | 0.132 |
| **Vital signs on day 1** |  |  |  |  |
| Heart rate, bpm | 98(84,112) | 97(83,111) | 99(85,112) | 0.518 |
| Systolic blood pressure, mmHg | 110(98,128) | 112(99,128) | 108(95,129) | 0.141 |
| Diastolic blood pressure, mmHg | 59(51,68) | 60(51,68) | 58(47,66) | 0.110 |
| Mean arterial pressure, mmHg | 76(66,88) | 76(67,90) | 75(60,86) | 0.068 |
| Respiratory rate | 20(16,25) | 19(15,24) | 21(16,26) | 0.008 |
| Body temperature, ℃ | 36.6(35.8,37.3) | 36.7(35.9,37.5) | 36.4(35.8,37.1) | 0.013 |
| SpO_2_, % | 98(95,100) | 98(96,100) | 97(94,100) | 0.015 |
| **Laboratory findings on day 1** |  |  |  |  |
| White blood cell, ×10^3^/uL | 12.2(7.6,17.6) | 12.3(7.2,17.8) | 12(8.4,17.0) | 0.823 |
| Neutrophil,% | 80.0(66.0,87.8) | 80.2(68.0,87.0) | 79.0(59.2,87.9) | 0.454 |
| Lymphocyte,% | 7.1(4.0,12.7) | 6.8(3.3,11.0) | 8.0(4.5,15.5) | 0.106 |
| Red blood cell distribution width | 15.9(14.6,18.2) | 15.3(14.3,17.7) | 16.6(15.3,19.6) | <0.001 |
| Platelets, ×10^3^/uL | 168(100,244) | 168(99,240) | 167.5(102.3,244.0) | 0.716 |
| Hematocrit, % | 31.6(26.8,36.6) | 31.3(26.1,36.1) | 31.9(27.6,36.9) | 0.235 |
| Hemoglobin,g/dL | 10.3(8.9,12.0) | 10.3(8.9,12.1) | 10.3(9.0,11.7) | 0.754 |
| D_dimer,ng/mL | 3850(2373,6408) | 3541.0(2526.5,6089.5) | 4058.5(2041.3,6607.5) | 0.799 |
| Prothrombin Time,s | 16.8(14.9,20.0) | 16.6(14.9,19.1) | 17.1(15.0,22.0) | 0.084 |
| International normalized ratio  Partial thromboplastin time,s | 1.7(1.4,2.3)  37.8(30.8,50.8) | 1.7(1.4,2.0)  37.4(30.9,49.4) | 1.7(1.4,2.6)  39.0(30.8,53.4) | 0.212  0.714 |
| Fibrinogen,mg/dL | 241.5(169.0,406.8) | 242.0(161.8,408.3) | 241.5(175.3,385.5) | 0.751 |
| Alanine aminotransferase, U/L | 46(23,124) | 53.0(25.0,185.3) | 40(20.5,98.5) | 0.031 |
| Alkaline phosphatase, U/L | 117.0(70.0,226.0) | 99.0(61.0,217.5) | 148.5(78.3,264.0) | 0.017 |
| Aspartate aminotransferase, U/L | 80.5(36.8,213.0) | 78.5(38.5,289.5) | 81.5(35.0,191.0) | 0.579 |
| Amylase,U/L | 48(28,77) | 43.5(26.8,72) | 53(31,88) | 0.082 |
| ABLI | 0.09(-0.04,0.22) | 0.12(0.01,0.23) | 0.05(-0.11,0.19) | 0.010 |
| Total bilirubin, mg/dL | 2.6(1.7,4.0) | 2.8(2.2,4.3) | 2.2(1.2,3.8) | <0.001 |
| Lactate dehydrogenaseU/L | 364.0(231.5,621.5) | 352.5(229.0,499.8) | 441.0(240.0,717.0) | 0.051 |
| Albumin,g/L | 2.7(2.2,3.1) | 2.7(2.4,3.1) | 2.6(2.1,3.1) | 0.068 |
| Blood urea nitrogen, mg/dL | 29.0(18.0,48.0) | 26.0(16.0,41.0) | 34.0(21.8,56.0) | <0.001 |
| Serum creatinine, mg/dL | 1.2(0.8,2.1) | 1.1(0.8,1.7) | 1.5(0.9,2.3) | <0.001 |
| Creatine phosphokinase,U/L | 108.5(50.3,290.0) | 112.5(50.8,296.0) | 105.5(50.3,) | 0.681 |
| Creatine kinase MB,U/L | 7(4,15) | 6.0(3.0,16.8) | 7.0(4.0,13.5) | 0.582 |
| Lactate,mmol/L | 2.9(1.8,5.6) | 2.6(1.7,5.0) | 3.6(2.3,6.7) | 0.001 |
| pH | 7.36(7.29,7.43) | 7.37(7.31,7.45) | 7.35(7.25,7.42) | 0.012 |
| pO_2 ,_mmHg | 114.0(78.5,205.0) | 131.5(86.3,266.3) | 104.0(71.0,199.0) | 0.031 |
| pCO2,mmHg | 37(32,45) | 38(34,44) | 37(28,47) | 0.262 |
| PaO_2_/FiO_2_ ratio | 218.0(125.0,347.0) | 237.0(144.6,360.6) | 175.5(101.8,334.0) | 0.049 |
| Base excess | -2.0(-8.0,1.0) | -1.0(-6.0,1.8) | -4.0(-9.0,0.0) | <0.001 |
| Anion gap | 17.0(13.0,20.0) | 16.0(13.0,19.0) | 18.0(14.0,21.5) | <0.001 |
| Bicarbonate, mmol/L | 21.0(18.0,25.0) | 22.0(19.0,25.0) | 20.0(17.0,25.0) | 0.078 |
| Serum calcium, mmol/L | 8.1(7.5,8.7) | 8.1(7.4,8.6) | 8.3(7.7,8.8) | 0.118 |
| Serum chloride, mmol/L | 104(100,109) | 105(101,110) | 102(97.8,109) | 0.008 |
| Serum sodium, mmol/L | 138(135,142) | 139(136,142) | 138(134,142) | 0.094 |
| Serum potassium, mmol/L | 4.2(3.7,4.7) | 4.2(3.7,4.6) | 43.(3.9,5.0) | 0.006 |
| Blood glucose, mg/dL | 123(100,158) | 121(102,154) | 126(95,168) | 0.677 |
| **Others** |  |  |  |  |
| Duration of ICU stay this time , day | 5.5(2.7,11.8) | 6.2(3.1,17.0) | 4.5(2.2,7.4) | <0.001 |
| **Urine** **output on day 1, mL** | 1232.5(578.2,2091) | 1419.0( 827.5,2419.0) | 895(328,1565) | <0.001 |
| **Severity of illness scores** |  |  |  |  |
| GCS  SIRS  SOFA  OASIS | 15(14,15)  3(3,4)  8(6,12)  37(30,44) | 15(14,15)  3(3,4)  8(5,10)  34(28,41) | 15(14,15)  4(3,4)  9(7,12)  41(34,46) | 0.042  0.019  <0.001  <0.001 |
| SAPSII | 47(36,59) | 40(33,53) | 54.5(44.0,63.0) | <0.001 |
| LODS | 7.0(4.0,9.0) | 6.0(4.0,8.0) | 8.0(5.8,10.0) | <0.001 |
| APSIII | 63(48,80) | 54.0(43.0,72.0) | 73(59.8,90.0) | <0.001 |

**S6 Table. Compare the performance evaluation of 8 machine learning classific-**

**ation models in predicting 28-day mortality rate in the external validation set.**

| **Classifiers** | **AUC** | **Accuracy (%)** | **Precision (%)** | | | **Recall** | | **Specificity (%)** | |
| --- | --- | --- | --- | --- | --- | --- | --- | --- | --- |
| **random forest** | 0.77 | 67.57 | 65.10 |  | 0.90 39.86 | | | |  |
| **classif.cv_glmnet**  **classif.svm** | 0.75  0.72 | 64.86  63.36 | 62.23  62.16 | | | 0.94  0.87 | 29.05  33.78 | |  |
| **classif.log_reg** | 0.74 | 61.56 | 60.67 | | | 0.88 | 29.05 | |  |
| **classif.lda** | 0.74 | 60.66 | 60.00 | | | 0.88 | 27.03 | |  |
| **classif.kknn** | 0.62 | 54.46 | 58.00 | | | 0.78 | 29.05 | |  |
| **Classif.xgboost** | 0.65 | 62.16 | 64.82 | | | 0.70 | 52.70 | |  |
| **classif.rpart** | 0.66 | 63.66 | 65.38 | | | 0.74 | 51.35 | |  |
| **AUC**: Area under curve. | | | | | | | | | |

**S7 Table. Compare performance evaluation of random forest and traditional disease severity scores in predicting 28-day mortality rate in the external**

**validation set.**

| **Model/scales** | **AUC** | **Accuracy(%)** | | **Precision(%)** | | **Recall** | **Specificity(%)** |
| --- | --- | --- | --- | --- | --- | --- | --- |
| **Random forest** | 0.77 | | 67.57 | 65.10 | 0.90 | | 39.86 |
| **Apsiii** | 0.61 | | 56.76 | 58.51 | 0.76 | | 32.43 |
| **Lods** | 0.62 | | 57.06 | 55.69 | 0.96 | | 8.11 |
| **Oasis** | 0.63 | | 61.56 | 60.44 | 0.89 | | 27.03 |
| **Sapsii** | 0.67 | | 64.56 | 65.16 | 0.78 | | 47.97 |
| **Sirs** | 0.58 | | 55.56 | 55.56 | 1.00 | | 9.46 |
| **Sofa** | 0.60 | | 57.96 | 57.19 | 0.97 | | 25.58 |
